# Essential oils as potential insecticides and behavior-modifying agents against *Bactrocera tryoni* (Diptera: Tephritidae)

**DOI:** 10.1093/jisesa/ieaf073

**Published:** 2025-10-06

**Authors:** Md Sahadat Hossain, Sanjana Akter, Md Forhad Hossain, Syed Zulfiqar Rizvi, Vivian Mendez, Phillip Taylor, Soo Jean Park

**Affiliations:** Applied BioSciences, Macquarie University, Sydney, New South Wales, Australia; Applied BioSciences, Macquarie University, Sydney, New South Wales, Australia; Applied BioSciences, Macquarie University, Sydney, New South Wales, Australia; Applied BioSciences, Macquarie University, Sydney, New South Wales, Australia; Applied BioSciences, Macquarie University, Sydney, New South Wales, Australia; Applied BioSciences, Macquarie University, Sydney, New South Wales, Australia; Applied BioSciences, Macquarie University, Sydney, New South Wales, Australia

**Keywords:** bioinsecticide, topical toxicity, repellency, oviposition deterrence, pest management

## Abstract

The Queensland fruit fly (Q-fly) *Bactrocera tryoni* is the most economically destructive tephritid pest in eastern Australia, inflicting substantial damage to diverse fruit and vegetable crops. Broad-spectrum, persistent, synthetic insecticides have been used to manage tephritid fruit flies. However, the adverse effects of these insecticides on human health, the environment, and nontarget organisms, as well as regulatory restrictions, have prompted the search for alternative control methods. This study explores the potential of essential oils as alternatives by evaluating their toxicity and behavior-modifying properties against adult Queensland fruit flies. We evaluated 16 essential oils for contact and fumigation toxicity, oviposition inhibition, and repellence. The chemical profiles of the essential oils were analyzed with Gas Chromatography-Mass Spectrometry and antennal responses were assessed by gas chromatography-electroantennographic detection. Chamomile, lemon-scented tea tree, and citronella exhibited notable contact toxicity (ED_50_ 0.054 to 0.068 mg/µl) after a 24-h exposure, while garlic, aniseed, pennyroyal, basil, and peppermint exhibited high fumigation toxicity (ED_50_ 3.293 to 4.950 µl/liter air) over the same period. Aniseed, cumin, and pennyroyal essential oils repelled both Queensland fruit fly sexes in 4-arm olfactometer assay. Aniseed, basil, chamomile, citronella, cumin, dill, garlic, lemon-scented tea tree, pennyroyal, peppermint, thyme, and yarrow essential oils inhibited oviposition. This study demonstrates essential oils as toxicants, oviposition deterrents and repellents, offering promising alternatives to conventional pest control methods for managing Queensland fruit fly populations.

## Introduction

The Queensland fruit fly (Q-fly), *Bactrocera tryoni* (Diptera: Tephritidae), is a highly destructive tephritid pest species across eastern Australia, responsible for severe damage to a wide variety of fruit and vegetable crops (Plant Health Australia 2008, Dominiak and Daniels 2012). Q-fly infests fruits of over 100 native and introduced host plants (Hancock et al. 2000, Oliver 2007), resulting in annual economic losses exceeding $300 million in Australia (DPIRD 2023). These losses include crop damage, management costs, and routine surveillance costs (DPIRD 2023). Q-fly is thought to be native to the tropical and subtropical coastal regions of Queensland and northern New South Wales in Australia (Froggatt 1911, Gurney 1910); however, over time, it has expanded its range to most of eastern Australia and has become established in several South Pacific island countries (Drew et al. 1978). The ability of Q-fly to spread by travelers as larvae in infested fruit (Dominiak et al. 2000) and to thrive in a wide range of climates is likely to lead to an expansion in the range of the species (Clarke et al. 2011, Popa-Báez et al. 2020).

Historically, the management of fruit fly pests has relied on cover sprays of broad-spectrum, persistent, synthetic insecticides such as organophosphates (Maklakov et al. 2001, Gazit and Akiva 2017), pyrethroids, and neonicotinoids (Senior et al. 2017). While these insecticides are cost-effective and offer efficient protection against fruit fly infestations, their usage presents chemical hazards to users and consumers, and commonly leads to adverse environmental impacts and harm to nontarget organisms (Fry 1995, Shukla et al. 2006, Goulson 2013). Overuse of synthetic insecticides has not only contributed to the development of insecticide resistance in many insects but has also negatively impacted beneficial natural enemies from production systems, resulting in pest resurgence (Gill and Garg 2014). To decrease the negative effects of synthetic insecticides, alternative pest management practices have been developed, including the use of different protein baits, sterile insect technique (SIT) and male annihilation technique (MAT) as part of integrated pest management (IPM) strategies for fruit fly control. Despite documented successes in the application of protein baits (McQuate et al. 1999, Patel et al. 2019, Nithya et al. 2023), SIT (Wong et al. 1992, Yamagishi et al. 1993, Ito and Kakinohana 1995, Plá et al. 2021) and MAT (Steiner et al. 1970, Koyama et al. 1984, Vargas et al. 2009, 2014), the techniques still face challenges related to cost effectiveness, cost recovery, technical difficulties, regional coordination, ethical and regulatory concerns, and the need for regular monitoring and surveillance.

Consequently, there is an ongoing demand for alternative approaches that reduce the reliance on synthetic insecticides and contribute to more sustainable pest management practices. One such approach involves using bioinsecticides that can function as softer toxicants, repellents, or oviposition deterrents when integrated with other IPM methods.

Bioinsecticides have gained prominence as an alternative to synthetic insecticides, with the global market valued at US $5.75 billion in 2022 (Vantage Market Research 2022). Due to their multi-faceted modes of action and higher biodegradability, bioinsecticides are considered environmentally safer and more sustainable than synthetic insecticides (Gupta and Dikshit 2010, Mehrotra et al. 2017, Borges et al. 2021, Ayilara et al. 2023, Hezakiel et al. 2024). This has led to increased interest in botanical insecticides, particularly essential oils (EOs), as potential tools for pest management (Ayvaz et al. 2010).

EOs are complex mixtures of 20 to 60-volatile and lipophilic compounds. Typically, they contain 2 or 3 major components in high concentrations, while other components are present in lower concentrations (Burt 2004, Bakkali et al. 2008). EOs are most commonly characterized by terpenes and terpenoids as their primary constituents (Modzelewska et al. 2005). However, they may also contain a variety of other compounds, including esters, amines, alcohols, aldehydes, phenols, ethers or oxides, heterocycles, amides, and ketones (Hüsnü et al. 2007, Tabanca et al. 2007). The diversity of these components contributes to the synergistic effects observed in EOs that enhance their efficacy as pest management agents (Miresmailli et al. 2006, Jiang et al. 2009, Martin et al. 2011, Pavela 2014).

Topical and fumigation assays have been employed to assess the toxicity of EOs against several tephritids. For example, rosemary (*Rosmarinus officinalis*), arborvitae (*Thuja occidentalis*), chan (*Hyptis suaveolens*), and lavender (*Lavandula angustifolia*) EOs exhibited both contact and fumigation toxicities against adult Mediterranean fruit flies (medfly) (*Ceratitis capitata*) (Benelli et al. 2012). Three citrus peel EOs, including eureka lemon (*Citrus limon*), yellow grapefruit (*Citrus paradise*), and navel orange (*Citrus × sinensis*), exhibited toxicities against Q-fly larvae (Muthuthantri et al. 2015). Peppermint (*Mentha piperita*) EO showed fumigation toxicity against Q-fly eggs, while broad-leaved peppermint (*Eucalyptus dives*) EO was toxic to both eggs and larvae (Hidayat et al. 2015). Pennyroyal (*M. pulegium*) (Mejdoub et al. 2019), and blue gum (*E. globulus*) EOs showed fumigation toxicity against olive fruit fly (*B. oleae*) (Bourakna et al. 2022). Exposure to volatiles from slash pine (*Pine slliottii*) and green propolis (*Baccharis dracunculifolia*) EOs resulted in lower longevity, survivorship and fecundity in South American fruit fly (*Anastrepha fraterculus*) and medfly adults (Oviedo et al. 2020). Additionally, carvacol and α-pinene, which are commonly found in Eos, exhibited fumigation toxicity against the oriental fruit fly (*B. dorsalis*) (Jaffar and Lu 2022).

In addition to toxicity, EOs have demonstrated potential as behavior-modifying agents. For instance, spotted wing drosophila (*Drosophila suzukii*) and common fruit fly (*D. melanogaster*) adults are repelled by various EOs or EO-based compounds, including mandarin (*Citrus reticulata*) and tea tree (*Melaleuca alternifolia*) (Bedini et al. 2020), peppermint (Renkema et al. 2016, Wang et al. 2021), hinoki cypress (*Chamaecyparis obtuse*) (Lee et al. 2015). Citral (mixture of geranial and neral) repelled melon fly (*Bactrocera cucurbitae*) (Zheng et al. 2021). Citronella grass (*Cymbopogon nardus*), copaiba (*Copaifera officinalis*), and clove (*Syzygium aromaticum*) repelled the medfly (Gonzaga et al. 2023). Furthermore, EOs can act as oviposition deterrents. Olive fruit flies were deterred from ovipositing in olives that had been treated with nano-emulsions of fennel (*Foeniculum vulgare*) and aniseed (*Pimpinella anisum*) EOs (Giunti et al. 2022). Mixtures and solutions of 6 essential oil compounds- (−)-*α*-pinene, (−)-*β*-pinene, (−)-α-bisabolol, carvacrol, (*R*)-(+)-limonene and thujone (α, β-mixture) sprayed on oranges were found to have concentration-dependent oviposition deterrence in oriental fruit fly (Jaffar and Lu 2022). EOs of black pepper (*Piper nigrum*) also deterred oviposition in oriental fruit fly and guava fruit fly (*B. correcta*) (Jaleel et al. 2020). Linalool, a commonly found constituent of EOs, had a deterrent effect on oviposition in medfly (Papanastasiou et al. 2020). Artificial fruits and apples sprayed with peppermint, lemon-scented tea tree (*Leptospermum petersonii*) and honey myrtle (*Melaleuca teretifolia*) EOs showed high repellency and oviposition-deterrent activities in Q-fly females (Hidayat et al. 2013). Although these results highlight the potential of EOs for managing tephritids, a broader screening of EOs is necessary to fully assess their efficacy. Despite extensive research on various EOs and their components against tephritids, there are currently no commercially available EOs specifically formulated for this purpose.

Evaluating EOs for their efficacy as toxicants, oviposition deterrents, or repellents for the control of adult Q-flies is important for the development of EOs as effective and sustainable pest management tools. A comprehensive understanding of the chemical composition of the tested EOs enhances this evaluation. Based on existing literature, we selected 16 EOs to explore their potential as control tools for adult Q-flies. The primary objectives of the study were (i) to assess the contact and fumigation toxicities of the selected EOs against Q-fly adults, (ii) to evaluate the effects of the EOs on the behavior of the Q-fly, and (iii) to establish the chemical basis of EO activity.

## Materials and Methods

### EOs and Chemicals

EOs of tea tree, pennyroyal, citronella grass, basil (*Ocimum basilicum*), lemon-scented tea tree, thyme (*Thymus vulgaris*), peppermint, aniseed, and yarrow (*Achillea millefolium*) were purchased from Tanah Essential Oil Company, Australia. EOs of garlic (*Allium sativum*) and dill (*Anthum sowa*) were purchased from New Directions Australia Pty Ltd EOs of cumin (*Cuminum cyminum*), davana (*Artemisia pallens*) and chamomile (*Matricaria chamomilla*) were purchased from Ahimsa Oils Pty Ltd, Australia. EO of river red gum (*Eucalyptus camaldulensis*) was purchased from Saysons Marketing Services (SMS), United States. EO of java citronella (*Cymbopogon winterianus*) was purchased from Buckley & Phillips, Australia. All the EOs purchased from various suppliers were described as ≥99.9% pure and natural in the product descriptions. Dimethyl sulfoxide (DMSO) (≥99.9%), acetone (≥99.5%), sabinene (≥75%), α-pinene (≥98%), β-pinene (≥ 99%), β-myrcene (≥90%), α-phellandrene (75%), (-)-α-thujone (≥96%), o-cymene (≥98%), p-cymene (≥98%), d-limonene (≥97%), eucalyptol (≥99%), diallyl disulfide (≥99%), γ-terpinene (97%), linalool (≥97%), citronellal (≥95%), (+)-2-bornanone (≥97%), isoborneol (≥95%), l*-*menthone (≥96%), *l-*menthol (≥99%), 1-terpine-4-ol (≥95%), citral (≥95%), 4-allylanisole (≥98%), *d-*carvone (≥96%), *p-*methoxyaldehyde (≥99.9%), pulegone (≥95%), geranial (≥97%), thymol (≥98.5%), menthyl acetate (≥97%), citronellyl acetate (≥95%), geranyl acetate (≥97%), and diethyl phthalate (≥99.5%) were purchased from Merck Life Sciences (Missouri, United States). Linalyl acetate (≥95%), α-terpineol (≥95%), neryl acetate (≥95%), isobornyl acetate (≥90%), β-caryophyllene (≥90%), and isopropyl myristate (≥98%) were purchased from TCI (Tokyo, Japan). α-Farnesene (≥95%) was purchased from BLDpharm (Shanghai, China).

### Rearing of Q-Fly

Q-flies were reared in a controlled environment room (CER) at 25 °C (±1 °C) temperature, 65% (±5%) relative humidity (RH), and a photocycle of light:dusk:dark:dawn (11.5:0.5:11.5:0.5 h). Eggs were collected from a colony which originated from the Central Coast region of New South Wales. A semi-transparent soft plastic bottle (300 ml) with approximately 100 holes (1 mm diameter) was used as an oviposition device. The oviposition device contained 20 ml of water to ensure adequate humidity and placed in a cage containing 14 to 20-d-old mature flies from 11:00 to 16:00 (light period) on a single day.

The inner wall of the oviposition device was washed thoroughly with reverse osmosis (RO) water and the water containing the eggs was transferred to a 50 ml Falcon tube. Once eggs settled to the bottom, water was decanted. A 250 µl of the egg suspension was transferred onto the surface of the larval diet. The larval diet container covered with plastic lids was left for 5 d. The lid of the container was then removed, and the larval diet container was transferred to a secondary container (12.5  liter, The Decor Corporation Pty. Ltd), which contained approximately 1 cm layer of vermiculite. The secondary container had 2 mesh-covered windows to provide ventilation (10 cm diameter). The third larval instar exited the diet and pupated in the vermiculite. The pupae were collected by sieving the vermiculite. Approximately 2,000 pupae were placed in a mesh cage (47.5 × 47.5 × 47.5 cm, MegaView Science Co., Ltd, Taiwan) for adult emergence. Adults were provided ad libitum with yeast hydrolysate (MP Biomedicals LLC, France), sugar, and RO water through a soaked wet sponge (Freudenberg Household Products Pty Ltd).

Larval diet was prepared following the method described by Moadeli et al. (2017) and Mainali et al. (2019) with minor modifications. Briefly, to prepare 1  liter of larval diet, brewer’s yeast (204 g) (Priority Health Pty Ltd), sugar (121.8 g), nipagin (2 g) (Sigma-Aldrich, Australia), sodium benzoate (2 g) (Sigma-Aldrich, Australia), citric acid (23.1 g) (Sigma-Aldrich, Australia), and RO water (500 ml) were blended to make a homogenous solution using a blender (Ninja Model BN750ANZ30, China). Agar (10 g) (Chem-Supply Pty Ltd, Australia) was mixed with RO water (500 ml) in a beaker (1 liter) and the mixture was microwaved for 4.5 min to reach boiling point. The boiled agar solution and canola oil (2 ml) (CRISCO, Australia) were then mixed with the other ingre­dients’ solution in the blender until homogenized. The hot gel diet (160 ml) was poured into plastic containers (17.2 × 12 × 3.9 cm, Chanrol Pty Ltd, Australia) and allowed to cool to room temperature.

### Contact Toxicity Assay

To prepare stock solutions of 20% (w/v) of EOs, 2.0 g of each EO was dissolved in 10 ml of DMSO. DMSO was selected to minimize solvent toxicity (Marino et al. 2019). These stock solutions were diluted as required in each experiment. In the initial screening, 14 to 17-d old flies (unsexed) in a plastic vial (7 ml) were chilled at 4 °C in a refrigerator for 4 to 5 min, and the immobile flies were then transferred into a Petri dish. 1 µl of 10% each EO in DMSO was applied to the dorsal thorax of a fly using a micropipette. A total of 20 adult Q-flies were tested for each EO. Only those EOs that induced at least 50% mortality within 24 h after exposure were selected for further investigation. Java citronella, citronella grass, lemon-scented tea tree, aniseed, yarrow, pennyroyal, thyme, and chamomile EOs met this criterion. Doses of 0.013, 0.025, 0.050, and 0.100 mg of EOs were applied on the dorsal thorax of chilled flies by pipetting 1 μl of 1.25%, 2.50%, 5.00%, and 10.00% (w/v) solutions, respectively. For each concentration, 4 replicates were performed, each consisting of 20 adult flies. Flies were taken from mixed-sex cages, and subsequent assays were conducted on flies of each sex separately to determine potential variations in toxicity between sexes. Control flies were treated with 1 µl of DMSO alone. Treated and control flies were introduced into a plastic cage (1.13  liter, Atlas Plastics Pty Ltd, Australia) that had a mesh screen (10.0 × 5.5 cm) on one side for ventilation. The cages were maintained under the same conditions as the insect rearing room. Flies were provided with a diet (sugar and solid yeast hydrolysate in separate Petri dishes), along with an agar water block (2 × 1 × 1 cm). Mortality was recorded at 6, 12, 24, and 48 h of exposure.

To prepare the agar water blocks, agar (20 g) was mixed with 1 liter of RO water in a beaker (2 liter) on a hot plate. The mixture was heated until it boiled, stirring constantly with a spoon to ensure homogeneity. The mixture was removed from the hotplate and allowed to cool to approximately 70 °C. The warm agar solution was poured into plastic containers to a thickness of 2 cm. After the agar mixture had cooled, the containers were covered with lids and stored in a refrigerator at 4 °C until used. The agar blocks were cut immediately before use.

### Fumigation Toxicity Assay

Fumigation toxicities of EOs were conducted using 500 ml glass jars (Rowe Scientific Pty Ltd, New South Wales, Australia) containing 20 males or females of 14 to 17-d old Q-fly, taken from mixed-sex cages to determine potential variations in fumigation toxicity between sexes. Five microlitres of each EO was directly pipetted to 1 cm^2^ of Whatman filter paper to give 10 µl/liter air concentration. The filter paper was wrapped with 2 layers of sterile gauze (Livingstone International Pty Ltd, Australia) to prevent flies from contacting the EO and placed inside the jar. Food (sugar, yeast hydrolysate) and an agar water block (1 cm^3^) were placed inside the jar, and the screw cap was then tightly sealed. Mortality was recorded after 24 h of exposure, and further testing was conducted based on the same selection criteria used in the contact toxicity assay. All EOs, except chamomile and davana, induced 50% or higher mortality in both sexes and were selected for further testing. The subsequent assays followed the same method as the initial screening, but with the EOs at different concentrations. Filter papers were treated with 0.63, 1.25, 2.50, and 5.00 µl of an EO to achieve concentrations of 1.25, 2.50, 5.00, and 10.00 µl/liter air to be used as treatments, and an untreated filter paper was used as control. The experimental jars were maintained under the same CER settings as for fly rearing (25 ± 1 °C, 65 ± 5% RH). Four replicates were collected from each group of flies. Mortality in each group was assessed after 6, 12, 24, and 48 h of exposure.

### Four-Arm Olfactometer Bioassay

**Fig. 1.**
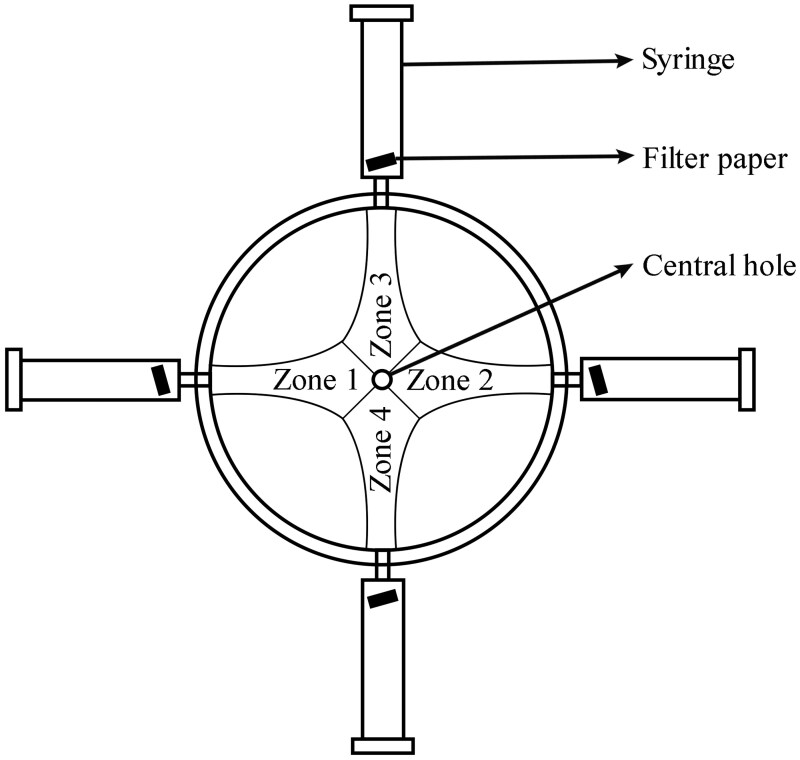
Diagram of the 4-arm olfactometer setup. Air was drawn through the central hole, connected in series to a flowmeter and a pump. The airflow rate was regulated by the flowmeter.

Acrylic 4-arm olfactometers (120 mm diameter) were used to evaluate the behavioral response of 14 to 17-d old male and female Q-fly to the EOs following the procedure described by Kempraj et al. (2022) with minor modifications (Fig. 1). Before each replication, olfactometers were cleaned using a nonionic detergent solution (5.0% Extran aqueous solution, w/v), washed with warm tap water and ethanol, and allowed to air dry overnight. The assays were conducted in a CER (25 ± 1 °C, 65 ± 5% RH). A filter paper (Whatmann No. 1, 70 mm diameter) was positioned at the center of the floor of the olfactometer to provide traction for the flies. Consistency of light around the olfactometers was achieved by positioning white LED lights in addition to the permanent lights of the CER. The 2 treated arms were at opposite sides of the olfactometer, and each arm had airflow over a filter paper strip (1 cm^2^) treated with 5 µl of EO solution (1.0%, w/v), while the 2 control arms, also opposite each other, had airflow over filter paper strips treated only with solvent (acetone). A single fly was introduced into an olfactometer through a central hole, and each fly was allowed a 5-min acclimation period. Air was pulled through the olfactometer using an air-pump system (Fig. 1), in which air was drawn through the central hole and passed in series through a flowmeter and a pump. The airflow rate was regulated by the flowmeter and maintained at 200 ml min^−1^. Fly locomotion was recorded for 10 min using a digital video camera (Panasonic HDC-HS700). To avoid directional bias, the olfactometer was rotated by 90° between assays. Thirty-two replications were conducted for each EO. Time spent in each zone of the olfactometer was analyzed using LoliTrack version 5 video tracking and behavior analysis software. The video analysis involved 2D tracking, which included calibrating distances, masking out unwanted areas, defining threshold images with the targeted object (fly), and tracking the movements of the fly. Data on the selected parameters (number of entries in each zone and the time spent in each entry) from the plotted control and treatment zones were extracted from the analysis.

### Oviposition Assay

Effects of EOs on oviposition were evaluated following Kempraj et al. (2019). An oviposition stimulant, γ-octalactone (Kempraj et al. 2019), was used as a positive control. Agarose plates were treated with γ-octalactone (2 µl of 0.05%, v/v hexane) alone, as the control, or blended with an EO solution (2 µl of 0.5%, v/v hexane) as treatment. To prepare the agarose plates, agar (4.0 g) was mixed with RO water (500 ml) in a 2 L beaker, which was microwaved until the contents were homogenized. The agar solution (0.8%, w/v) was cooled to approximately 60 °C, and 20 ml of this solution was poured into 50 ml plastic vials. The mixture was transferred into pre-cooled Petri dishes on ice and kept at 0 °C with lids capped until the agarose gel solidified. The agar plates were prepared freshly as required. The oviposition plates, which contained either γ-octalactone alone (control) or γ-octalactone in combination with an EO, were placed in different cages (47.5 × 47.5 × 47.5 cm, BugDorm-4S4545), each housing 50 gravid females (13 to 17-d old). Mating activities were observed before each experiment to ensure females were gravid in the mixed sex cages. Cages with control and treated oviposition plates were kept in separate CERs to ensure that the control cage remained unaffected by any odors emanating from the treatment. The assay was carried out for 24 h, and the eggs laid in both plates were counted under a stereoscope (Olympus SZX 12, Japan). ­Thirty-two replications were run for each EO.

### GC-MS Analysis

Gas chromatography mass spectrometry (GC-MS) was conducted using a Shimadzu GCMS-NX2030 spectrometer equipped with a split injector and HS Rtx-5MS (30 m × 0.25 mm ID × 0.25 μm film) fused silica capillary column and integrated single quadrupole mass spectrometer (Shimadzu Corporation, Kyoto, Japan). The carrier gas was Helium (He) (99.999%) (BOC, North Ryde, New South Wales, Australia) at a flow rate of 1.5 ml min^−1^.

A stock solution of each EO was prepared by dissolving a known amount of each EO in 10 ml of hexane (4.0 to 10 mg/ml, w/v). The stock solution was diluted to give the EO sample concentrations of 20 to 50 μg/ml (w/v). An aliquot of 1 µl of sample was injected at split mode (20:1 ratio) where the injector temperature was 250 °C. The oven temperature ­program was: 60 °C (1 min), increased to 270°C at a rate of 15 °C/min, and increased to 300 °C at a rate of 30 °C/min and held for 3 min. The ion source and transfer line temperatures were 230 and 250 °C, respectively. The ionization method was electron impact at a voltage of 70 eV. The spectra were obtained over a mass range of m/z 35 to 650 at scan mode. The data were analyzed through Shimadzu GCMS Postrun software (V4.52). Mass spectra were compared with the NIST library (NIST17-1, NIST17-2, NIST17s) to identify related compounds. Fragmentation patterns and retention indices available in the literature were used to suggest candidate compounds. The suggested compounds were purchased and analyzed by GC-MS. Identification of a compound was confirmed by comparing the retention time and fragmentations of the genuine compound. The solvent, *n-*hexane, was regularly subjected to GC-MS runs to detect any impurities.

### Electrophysiological Assay

Gas chromatography-electroantennographic detection (GC-EAD) was carried out to identify the compound(s) of EOs that were detected by antennae and were hence strong candidates as active in repellence and oviposition deterrence. The system consisted of a coupled system of Agilent GC 7890 (Agilent, California, United States) and Syntech electrophysiological recording equipment (Syntech, Hilversum, The Netherlands). GC system was equipped with a split/splitless injector, SH Rtx-5MS (30 m × 0.25 mm, 0.25 µm film) fused silica capillary column (Shimadzu Corporation, Kyoto, Japan) and a flame ionization detector (FID). The carrier gas was hydrogen (99.999%) (BOC, North Ryde, New South Wales, Australia) at a flow rate of 2.5 ml/min. An aliquot of 1 μl (5 mg/ml, w/v) of EO sample was injected at split mode (50:1 ratio), where the injector temperature was 270 °C. The oven temperature program is as follows: 50 °C (1 min), increased to 260 °C at 15 °C/min and held for 5 min. Effluent from GC was split, and the split ratio directed to FID and electrophysiological detector was 1:1.5. Two microelectrodes, consisting of Ag (silver) wire and micro glass tube, were filled with electroconductive gel (Spectra 360, Parker Laboratories Inc., United States). The head of a live male or female Q-fly was separated from the body using ophthalmic surgery micro scissors (Australian Entomological Supplies Pty Ltd). The base of a fly head was affixed to a small amount of gel (Parker Laboratories, Inc., United States) protruding from the micro glass tube at the end of one of the recording electrodes, while the tips of the fly antenna were slightly inserted in the gel coming out of the micro glass tube of the other recording electrode. The signals were passed through a high impedance amplifier (IDAC4, Syntech, Hilversum, The Netherlands). The outputs from the electrophysiological amplifier and the FID were monitored at the same time and analysed using GC-EAD 2014 (V1.2.5). The identification of FID peaks was confirmed by GC-MS, running at the same GC conditions, including the column type, as the GC-EAD system.

### Statistical Analysis

Mortality data from contact and fumigation toxicity assays were analyzed using a probit model with SPSS software. The number of killed flies at each observation time was analysed with a Poisson generalized linear model (GLM), utilizing R software (version 4.4.2) to determine the effects of EO, sex, time, dose, and the interactions between EO, sex, time, and dose. The data from the repellence assay were analysed using Wilcoxon’s test. The oviposition assay data were first normali­zed using the Yeo-Johnson transformation and later analysed using a *t*-test. Raw data from both the olfactometer and oviposition assays were used to generate graphical illustrations.

## Results

### Contact Toxicity Assay

Table 1 lists the ED_50_ doses of 8 EOs that caused 50% mortality to male and female Q-flies in contact toxicity assays. Chamomile and citronella EOs showed the highest toxicity overall (ED_50_ 0.054 to 0.066 mg/µl after 24 h of exposure). Lemon-­scented tea tree, aniseed, yarrow, pennyroyal, and thyme also showed contact toxicity with an ED_50_ range of 0.055 to 0.087 mg/µl after 24 h exposure. A Poisson GLM analysis showed contact toxicity differed significantly among the tested EOs for both sexes of the Q-fly, with χ^2^_(8,1143)_ = 1570.93, *P *< 0.0001. Significant interactions were observed between EOs and dose (χ^2^_(8,1115)_ = 68.62, *P *< 0.0001), as well as between EOs, time, and dose (χ^2^_(1,1142)_ = 27.03, *P *< 0.0001). No other interactions were detected.

**Table 1.** Contact toxicity assays

Essential oil	Fly	ED_50_ (mg/µl)	LCL	UCL	SE
Java citronella	Male	0.058	0.045	0.072	0.077
	Female	0.062	0.051	0.075	0.076
Citronella grass	Male	0.055	0.043	0.068	0.075
	Female	0.066	0.054	0.079	0.077
Lemon-scented tea tree	Male	0.055	0.043	0.069	0.076
	Female	0.068	0.056	0.081	0.077
Aniseed	Male	0.071	0.058	0.085	0.080
	Female	0.076	0.063	0.089	0.080
Yarrow	Male	0.072	0.058	0.086	0.081
	Female	0.087	0.074	0.101	0.083
Pennyroyal	Male	0.075	0.062	0.090	0.081
	Female	0.076	0.064	0.090	0.080
Thyme	Male	0.074	0.060	0.088	0.082
	Female	0.085	0.072	0.100	0.083
Chamomile	Male	0.054	0.041	0.067	0.076
	Female	0.065	0.053	0.077	0.077

ED_50_ values and 95% confidence limits of EOs in male and female Q-fly after 24 h exposure. The values in mg represent the delivered amount of an EO when an aliquot of 1 μl of EO solution was applied on the dorsal thorax of adult Q-flies.

LCL, lower confidence level; UCL, upper confidence level; SE, standard error.

### Fumigation Toxicity Assay

Table 2 presents the ED_50_ values of EOs for both sexes from the fumigation toxicity assays conducted on both sexes of Q-fly. ED_50_ values of EOs varied across different exposure times: 5.774 to 10.741 µl/liter air after 6 h, 3.830 to 8.775 µl/liter air after 12 h, 3.293 to 7.258 µl/liter air after 24 h, and 2.476 to 6.336 µl/liter air after 48 h. Garlic exhibited the highest toxicity in both sexes of Q-fly. Aniseed, basil, pennyroyal and peppermint displayed higher fumigation toxicities with ED_50_ values ranging from 3.593 to 4.950 µl/liter after 24 h exposure. Thyme, dill, river red gum, and lemon-scented tea tree showed moderate fumigation toxicity (ED_50_ 3.974 to 5.243 µl/liter after 24 h exposure). In contrast, cumin, yarrow, java citronella, citronella grass, and tea tree exhibited low fumigation toxicities (ED_50_ 5.079 to 7.258 µl/liter at 24 h of exposure). In parallel, a Poisson GLM analysis was conducted to determine the effects of EO type, sex, dose, and time on mortality. The analysis revealed mortality differed significantly among the tested EOs for both sexes, with χ^2^_(14,1905)_ = 2438.8 and *P *< 0.0001. A significant difference in mortality between sexes was observed ( χ^2^_(14,1888)_ = 119.5, *P *< 0.0001). Significant interactions were found between EOs and doses ( χ^2^_(14,1859)_ = 211.1, *P *< 0.0001); between time and dose ( χ^2^_(1,1857)_ = 66.1, *P *< 0.0001); and among EOs, sexes and doses ( χ^2^_(14,1829)_ = 35.4, *P *< 0.001). No other interactions were observed.

**Table 2.** Fumigation toxicity assays

Essential oil	Fly	ED_50_ (µl/liter air)	LCL	UCL	SE
Aniseed	Male	3.593	2.614	4.577	0.077
	Female	4.308	3.403	5.224	0.080
Basil	Male	4.267	3.271	5.276	0.078
	Female	4.728	3.806	5.664	0.082
Citronella grass	Male	7.077	5.995	8.189	0.089
	Female	5.451	4.510	6.41	0.084
Cumin	Male	6.506	5.449	7.589	0.087
	Female	5.079	4.157	6.017	0.082
Dill	Male	5.820	4.786	6.877	0.083
	Female	4.339	3.426	5.264	0.081
Garlic	Male	3.293	2.316	4.272	0.076
	Female	3.314	2.429	4.203	0.077
Java citronella	Male	7.258	6.184	8.363	0.088
	Female	5.377	4.463	6.306	0.083
Lemon-scented tea tree	Male	6.151	5.128	7.198	0.082
	Female	4.895	3.974	5.832	0.081
Pennyroyal	Male	4.744	3.732	5.772	0.081
	Female	4.846	3.910	5.798	0.083
Peppermint	Male	4.878	3.868	5.903	0.081
	Female	4.950	4.030	5.885	0.082
River red gum	Male	5.491	4.472	6.531	0.083
	Female	6.231	5.243	7.242	0.089
Tea tree	Male	7.178	6.074	8.313	0.091
	Female	6.526	5.560	7.514	0.089
Thyme	Male	5.828	4.773	6.908	0.083
	Female	5.132	4.198	6.083	0.083
Yarrow	Male	7.130	6.038	8.253	0.089
	Female	6.882	5.875	7.913	0.092

ED_50_ values and 95% confidence limits of EOs in male and female Q-fly after 24 h exposure. The values in μl/liter air represent the concentration of the delivered amount of an EO in a glass bottle.

LCL, lower confidence level; UCL, upper confidence level; SE, standard error.

### Four-Arm Olfactometer Bioassays

#### Number of Entries in the Olfactometer Zones

**Fig. 2.**
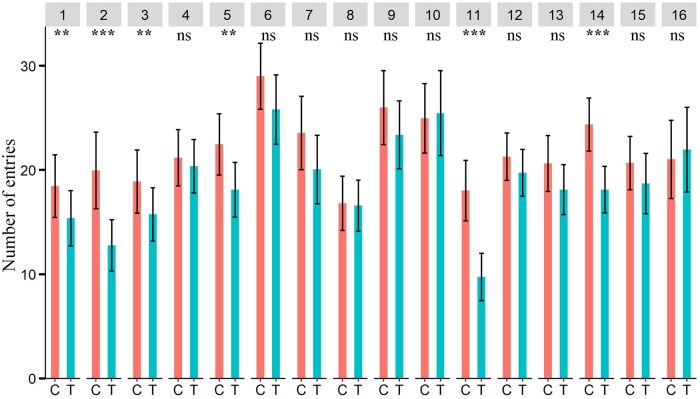
Four-arm olfactometer bioassays. Number of entries of Q-fly males in control and EO-treated zones. EOs: (1) aniseed, (2) basil, (3) chamomile, (4) citronella grass, (5) cumin, (6) davana, (7) dill, (8) garlic, (9) java citronella, (10) lemon-scented tea tree, (11) pennyroyal, (12) peppermint, (13) river red gum (14) tea tree (15) thyme, and (16) yarrow; Degrees of freedom of all tested EOs: 1, 64. The asterisks indicate the level of significance (^***^P < 0.001; ^**^P < 0.01; ^*^P < 0.05; ^ns^nonsignificant *P *≥ 0.05) for each EOs; C and T indicate control and treatment, respectively.

**Fig. 3.**
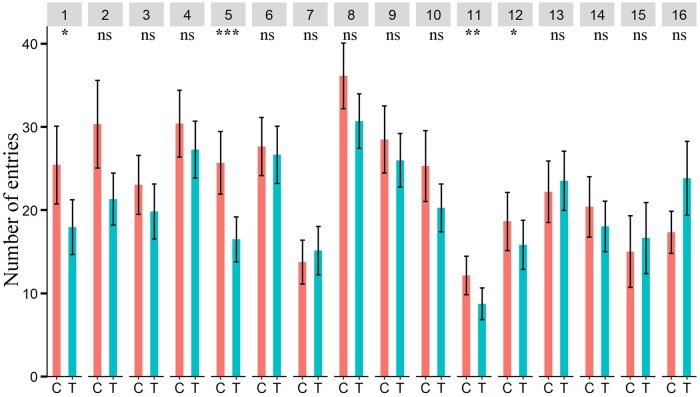
Four-arm olfactometer bioassay. Number of entries of Q-fly females in the control and EO-treated zones. EO: (1) aniseed, (2) basil, (3) chamomile, (4) citronella grass, (5) cumin, (6) davana, (7) dill, (8) garlic, (9) java citronella, (10) lemon-scented tea tree, (11) pennyroyal, (12) peppermint, (13) river red gum (14) tea tree (15) thyme, and (16) yarrow; Degrees of freedom of all tested EOs: 1, 64. The asterisks indicate the level of significance (^***^P < 0.001; ^**^P < 0.01; ^*^P < 0.05; ^ns^nonsignificant *P *≥ 0.05) for each EOs; C and T indicate control and treatment, respectively.

The number of entries of male Q-fly in the olfactometer zones treated with aniseed, basil, chamomile, cumin, pennyroyal, and tea tree EOs was significantly lower than in the control zones (Fig. 2), while the number of entries of female Q-fly in zones of the olfactometer treated with aniseed, cumin, pennyroyal and peppermint EOs was significantly lower than in the control zones (Fig. 3).

#### Time Spent in the Olfactometer Zones

**Fig. 4.**
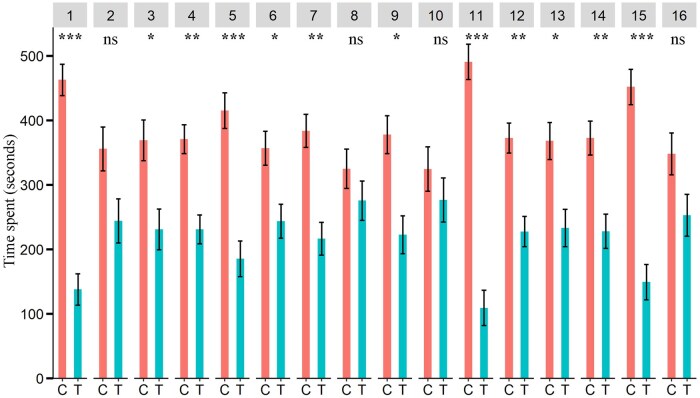
Four-arm olfactometer bioassay. Time spent (s) by Q-fly males in the control and EO-treated zones. EO: (1) aniseed, (2) basil, (3) chamomile, (4) citronella grass, (5) cumin, (6) davana, (7) dill, (8) garlic, (9) java citronella, (10) lemon-scented tea tree, (11) pennyroyal, (12) peppermint, (13) river red gum (14) tea tree (15) thyme, (16) yarrow; degrees of freedom of all tested EOs: 1, 64. The asterisks indicate the level of significance (^***^P < 0.001; ^**^P < 0.01; ^*^P < 0.05; ^ns^nonsignificant *P *≥ 0.05) for each EOs; C and T indicate control and treatment, respectively.

**Fig. 5.**
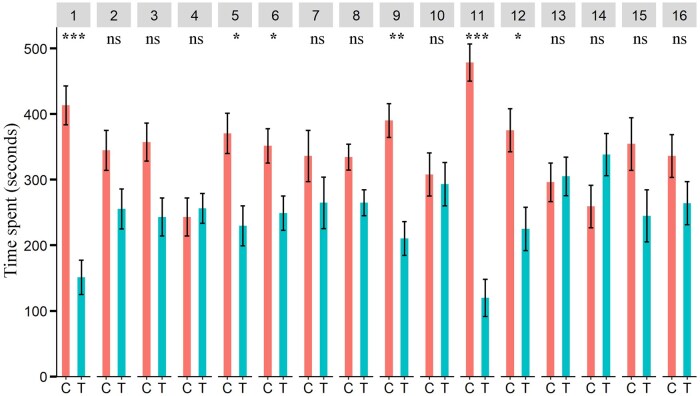
Four-arm olfactometer bioassay. Time spent (sec) by Q-fly females in the control and EO-treated zones. EO: (1) aniseed, (2) basil, (3) chamomile, (4) citronella grass, (5) cumin, (6) davana, (7) dill, (8) garlic, (9) java citronella, (10) lemon-scented tea tree, (11) pennyroyal, (12) peppermint, (13) river red gum, (14) tea tree, (15) thyme, and (16) yarrow; Degrees of freedom of all tested EOs: 1, 64. The asterisks indicate the level of significance (***P < 0.001; **P < 0.01; *P < 0.05; ^ns^nonsignificant *P *≥ 0.05) for each EOs; C and T indicate control and treatment, respectively.

Male Q-fly spent less time in the olfactometer zones treated with aniseed, chamomile, citronella EOs, cumin, davana, dill, pennyroyal, peppermint, river red gum, tea tree and thyme than in the control zones (Fig. 4) while females spent less time in the zones treated with aniseed, cumin, davana, java citronella, pennyroyal and peppermint (Fig. 5). Additionally, average time per visit for males and females based on the same dataset is presented in Supplementary Figs S1 and S2, respectively.

### Oviposition Assay

**Fig. 6.**
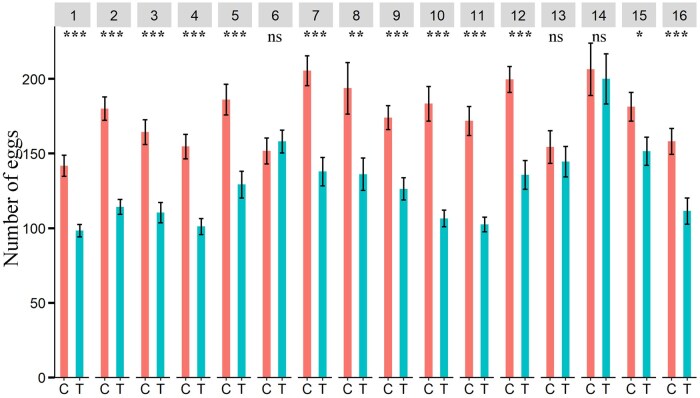
Oviposition assay. Number of eggs laid by Q-fly females in control and EO-treated agarose plates. EO: (1) aniseed, (2) basil, (3) chamomile, (4) citronella grass, (5) cumin, (6) davana, (7) dill, (8) garlic, (9) java citronella, (10) lemon-scented tea tree, (11) pennyroyal, (12) peppermint, (13) river red gum (14) tea tree (15) thyme, and (16) yarrow; Degrees of freedom of all tested EOs: 1, 64. The asterisks indicate the level of significance (***P < 0.001; **P < 0.01; *P < 0.05; ^ns^nonsignificant *P *≥ 0.05) for each EOs; C and T indicate control and treatment, respectively.

Female Q-fly laid significantly fewer eggs in oviposition substrates treated with aniseed, basil, chamomile, citronella EOs, cumin, dill, garlic, lemon-scented tea tree, pennyroyal, peppermint, thyme and yarrow EOs compared to those in the control substrates (Fig. 6).

### GC-MS Analysis

In GC-MS analysis, monoterpenes, monoterpenoids, and sesquiterpenes were the most common chemical classes found in EOs (Supplementary Table S3). *p*-Cymene, β-myrcene, d-limonene, linalool, α-pinene, β-pinene were found in all the studied EOs, except davana, garlic, and chamomile. Major compounds found in EOs are citronellal in citronella EOs, d-limonene in dill, menthone in peppermint, cuminaldehyde in cumin, geranial in lemon-­scented tea tree, 1-terpinen-4-ol in tea tree, (*E*)-anethole in aniseed, eucalyptol in yarrow and river red gum, pulegone in pennyroyal, davanone in davana, thymol in thyme, linalool in basil, diallyl disulphide in garlic, β-farnesene in chamomile.

### Electrophysiological Assay Results

**Fig. 7.**
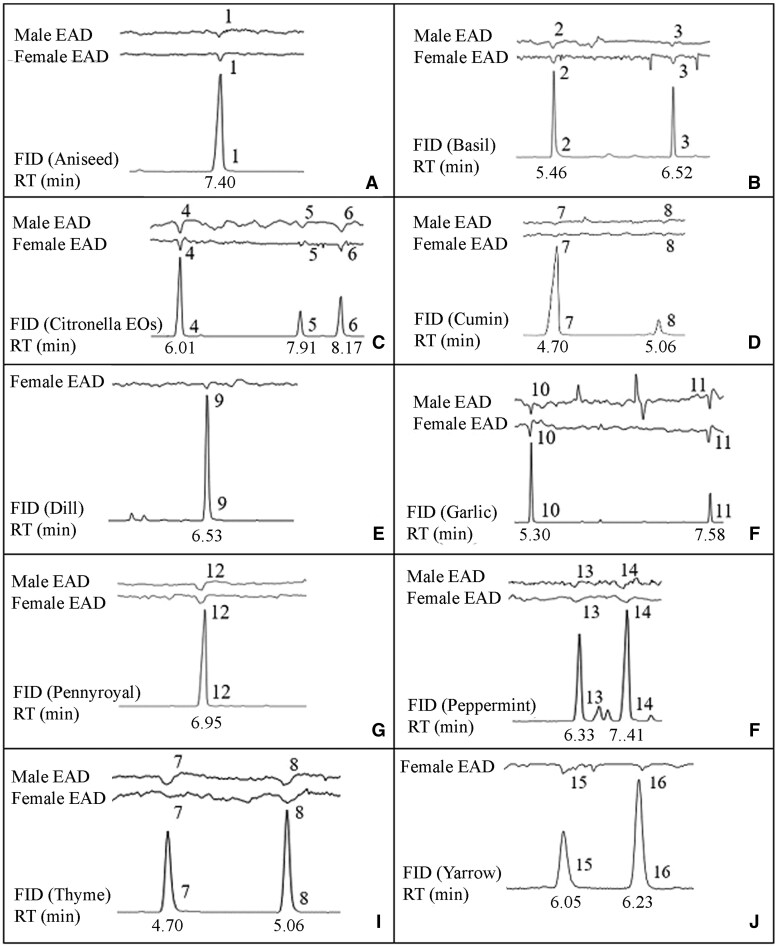
FID response and EAD of Q-fly males and females to EOs- (a) aniseed, (b) basil, (c) citronella EOs, (d) cumin, (e) dill; (f) garlic, (g) pennyroyal, (h) peppermint, (i) thyme, and (j) yarrow; numbered peaks indicate EAD-active compounds: (*E*)-anethole (1); linalool (2); estragole (3); citronellal (4); neral (5); geranial (6); *p*-cymene (7); *γ*-terpinene (8); *d-*carvon (9); diallyl disulfide (10); diallyl trisulfide (11); pulegone (12); *l*-menthol (13); menthone (14); (+)-2-bornanone (15); and isoborneol (16).

The results of GC-EAD assays of male and female Q-fly are presented in Fig. 7. Antennae of both male and female flies responded to (*E*)-anethole in aniseed; linalool and estragole in basil; citronellal, neral, and geranial in 2 citronella EOs; diallyl disulfide and diallyl trisulfide in garlic; pulegone in pennyroyal; menthone and l-menthol in peppermint; *p*-cymene and γ-terpinene in thyme and cumin EOs. Female antennae responded to *d*-carvon of dill; (+)-2-bornanone and isoborneol of yarrow EOs.

## Discussion

This study identified several EOs that have potential for development as new tools for the management of Q-fly. While citronella (java citronella and citronella grass), lemon-scented tea tree, and chamomile showed good contact toxicity, aniseed, basil, and garlic exhibited good fumigation toxicity. Aniseed, cumin and pennyroyal exhibited repellent effects against both sexes, while aniseed, basil, chamomile, citronella, cumin, garlic, lemon-scented tea tree, peppermint, thyme, and yarrow deterred oviposition. The identified chemical profiles of the tested EOs consistently suggest the certain constituents may be responsible for EOs’ contact and fumigation toxicities. Our electrophysiological study also identified compound(s) in each EO that may be responsible for repellent and/or oviposition-­deterrent activities.

Higher contact toxicities of citronella grass, java citronella, lemon-scented tea tree, and chamomile EOs against adult Q-flies have been demonstrated in our study. While contact toxicity of lemon-scented tea tree EO against the mite (*Tyrophagus putrescentiae*) has been established using a filter paper diffusion assay (Song et al. 2016), direct comparisons to our findings are challenging due to differences in assay methods. Chamomile EO has been tested in spotted wing drosophila and medfly but did not show significant toxicity (Barakat et al. 1985). The toxic effects of EOs are likely influenced by their major chemical constituents. For example, the prominent compound found in citronella EO was citronellal, which has also been found in lemon-scented tea tree EO, along with geranial and neral as its major compounds. These compounds have been found in orange peel EO as the major constituents and have shown contact toxicity against the rice weevil, (*Sitophilus ­oryzae*) (Mosa et al. 2022), although the assay method was different from our study. These compounds also showed strong contact toxicity to spotted wing drosophila (Jang et al. 2017). Chamomile EO, in contrast, contains sesquiterpenes, including β-farnesene and α-bisabolene isomers as its major constituents. Interestingly, β-farnesene is found in common wormwood (*Artemisia absinthium*) as the major constituent, and has shown toxic effects on 6 mosquito species (Govindarajan and Benelli 2016). Citronellal, geranial, and neral are all C10 based terpenoids, while β-farnesene and α-bisabolene isomers are sesquiterpene (C15). Despite differences in their chain length, all are composed of isoprene units and prone to atmospheric oxidation. These characteristics of the compounds may be relevant to the observed higher contact toxicity against Q-fly found in the present study. Our results suggest both C10-based terpenoids and sesquiterpenes (C15) found in the current study may have potential for further exploration to develop as toxicants.

Garlic EO has been extensively studied as a fumigant against various stored products and grain pests, including the maize weevil (*Sitophilus zeamais*) (Chaubey 2017), the grain moth (*Sitotroga cerealella*) (Yang et al. 2012), and flour beetle (*Tribolium* spp.) (Işıkber et al. 2009, Yang et al. 2010), demonstrating effective fumigation toxicities. Similarly, our results suggest strong potential for garlic EO as a fumigant. In our study, diallyl disulfide was identified as the predominant compound in garlic EO, likely contributing to its fumigant toxicity against adult Q-flies. This possibility is supported by previous studies on garlic EO and/or individual constituents of garlic EO against various insects. Yang et al. (2012) investigated garlic EO and its 2 major compounds, diallyl disulfide and diallyl trisulfide, on adult grain moths and observed significant fumigant toxicity. They reported LC_50_ values of 1.33, 0.99, and 1.02 μl/liter for garlic EO compounds, diallyl disulfide and diallyl trisulfide, respectively. Although these values are lower than that required for Q-fly in our study, they remain comparable, considering Q-fly and grain moths may exhibit different physiological responses. In addition to garlic EO, aniseed, basil, pennyroyal, and peppermint EOs also exhibited fumigation toxicities in our study. The major compounds in aniseed and basil, (*E*)-anethole and estragole, respectively, are structural isomers, suggesting an anisole motif with a propenyl side chain, which is common in (*E*)-anethole and estragole, potentially contributing to their fumigation toxicity. Likewise, pulegone, the major compound in pennyroyal, and menthone and menthol in peppermint, are closely related compounds containing cyclohexanone or cyclohexanol with methyl and isopropyl/isopropenyl side chains, which may also play a role in their fumigation toxicity. While our study presents initial findings on the fumigation toxicities of garlic, aniseed, basil, pennyroyal, and peppermint against adult Q-fly, it is important to note that further exploration of chemical information in EOs in the literature may provide additional candidates. Moreover, the development of EO blends as tools, rather than relying on single compounds, is crucial. A previous study (Feng and Isman 1995) demonstrated that botanical insecticides in a mixture of several active constituents delayed the development of resistance compared to the use of a single active ingredient.

The findings of this study indicate that the toxicity mechanism of the individual EO compounds may differ between contact and fumigation applications, possibly due to differences in routes of entry. Citronella, lemon-scented tea tree, and chamomile exhibited the highest contact toxicity but showed comparatively low fumigation toxicity. In contrast, garlic, aniseed, and basil were the 3 most toxic EOs in fumigation assays. The volatility of individual compounds in EOs does not fully explain the observed difference. For example, the retention indices of citronellal, a major compound in citronella EOs (contact toxicant) and diallyl disulfide, a major compound in garlic (fumigant), indicate their similar volatilities, yet citronella EOs were not effective fumigants. This discrepancy suggests that the characteristics of the compounds, such as structure, polarity, or interactions with biological targets, may influence their efficacy. Notably, the compounds effective in contact toxicity and fumigation belong to different chemical classes, with contact toxicant (e.g. citronellal) predominantly being C10 terpenoids and fumigants (e.g. diallyl disulfide) being sulfur-containing compounds. These findings highlight the importance of exploring the distinct chemical and physical properties that contribute to contact versus fumigation efficacy, providing information for targeted development of EO-based bioinsecticides.

In this study, EOs exhibited varying degrees of contact and fumigation toxicity. However, the ED_50_ values (Tables 1 and 2, and Supplementary Tables S1 and S2) obtained under the laboratory assays may not directly reflect effective or safe concentrations for field applications. Due to the diverse chemical compositions and mechanisms of action among different EOs, it is difficult to generalize an appropriate application dose across all tested EOs. Moreover, phytotoxicity remains a critical concern, as high EO concentrations can adversely affect plants, causing leaf damage, growth inhibition, or mortality (Kordali et al. 2008, Ibáñez and Blázquez 2020). Further investigation is essential to optimize EO-based insecticide formulations that maintain efficacy against target pests while minimizing phytotoxic risks and ensuring plant safety under field or protected cropping conditions.

Although EOs demonstrate contact and fumigation toxicities against Q-flies, their high volatility and stability issues pose challenges for use as toxicants in open fields. These characteristics can lead to rapid dissipation, degradation by sunlight, or oxidation, reducing their effectiveness. Appropriate formulation of EOs, such as microencapsulation and/or emulsification (Sousa et al. 2022), may help mitigate the issues with volatility and stability in field applications. Given the volatile characteristics of EOs, the use of EOs in protected cropping systems, such as greenhouses or netted enclosures, may also be more appropriate.

Citronellas and lemon-scented tea tree EOs have demonstrated both toxicity and oviposition-deterrent activity in Q-fly. While the repellent activities of EOs have been investigated extensively against mosquitoes (Erler et al. 2006, Nerio et al. 2010), this study is the first to report the repellent and oviposition-deterrent activities of the tested EOs on Q-fly, with the exception of lemon-scented tea tree and peppermint (Hidayat et al. 2013). A previous study found that lemon-scented tea tree and peppermint EOs did not repel Q-fly adults (Hidayat et al. 2013), a finding corroborated by our results. The present study also found citronella EOs to be an effective agent as both an oviposition deterrent and contact toxicant. Citronella EO has previously shown repellent activity against peach fruit fly (*B. zonata*) and medfly (Fouda et al. 2017). Similarly, Bothon et al. (2013) found that mated and unmated melon flies were repelled by ­citronella grass odor. This study is also the first to report oviposition-deterrent activities of aniseed, basil, chamomile, cumin, dill, garlic, thyme, and yarrow against Q-fly. Additionally, aniseed, basil, cumin, and pennyroyal demonstrated the repelling activities against females. These findings suggest that EOs, particularly aniseed, basil, cumin, and pennyroyal, could be integrated into a push-pull system when combined with attractants.

The behavioral responses of Q-fly females in olfactometer assays exhibit evidence of spatial deterrence by a number of EOs, as highlighted in Figs 4 and 5, and Supplementary Figs S1 and S2. Females showed reduced presence and spent less time in zones treated with aniseed, cumin, java citronella, pennyroyal, and peppermint EOs, indicating avoidance behavior toward the volatiles of these EOs. This pattern is consistent with the oviposition results, where significantly fewer eggs were laid on agarose plates treated with the same EOs. It is likely that the volatiles from EOs create a spatial barrier, disrupting the Q-fly’s host and oviposition site searching behaviors by interfering with olfactory cues.

Aniseed, java citronella, and pennyroyal EOs exhibit both spatial repellency and contact toxicity against Q-flies. This dual mode of action appears beneficial in terms of the overall bio-­efficacy of EOs; however, it also raises important considerations regarding practical effectiveness under field conditions. Spatial repellents act as behavioral barriers, deterring flies from approaching or settling on treated areas, thereby reducing the risk of pest infestation. In contrast, contact toxicants rely on direct interaction between the pest and the treated surface to deliver a lethal dose. Subsequently, strong repellent effects may inadvertently reduce contact rates, potentially lowering mortality associated with contact toxicity. Further investigation is required to determine which mode of action predominates under semi-field and field conditions to precisely assess real-world efficacy. Spatial repellents may be more suitable for enclosed or confined environments, whereas formulations intended to exploit contact toxicity may benefit from surface applications, such as treated fabrics or nets.

Increases in dose and exposure time significantly enhanced both contact and fumigation toxicities of EOs, indicating the toxicities were dose- and time-dependent. Similar trends have been widely observed in studies of other insects (Odeyemi et al. 2008, Ebadollahi et al. 2010). Penetration and absorption of higher amounts of EO compounds over time likely intensify the disruption of physiological functions in the flies. It highlights the importance of optimizing the application dose and exposure duration for effective management of Q fly populations. Interestingly, this study found that the sensitivity to fumigant application of EOs differs between sexes, with females being more susceptible than males. However, no sex-based differences were observed in sensitivity to topical applications. This pattern of sex-specific sensitivity to EOs has also been found in other insects (Kim et al. 2016). Differences in response to environmental stress (Teder and Kaasik 2023) may be the reason for showing sex differences in fumigation toxicity in this study.

We observed variation in the compositions of some of the EOs from previous studies (Supplementary Table S3). These compositional variations in EOs from the same plant species can arise from factors such as the geographical origin of the plants used to extract the oils, seasonal influences, atmospheric conditions, cultivation practices, and extraction methods ­(Muttalib et al. 2018, Majewska et al. 2019). Conducting chemical analysis of the studied EOs is necessary to describe the oils in particular experimental conditions, as variation in the composition will significantly influence the activity or efficacy of an EO.

We noted that female Q-fly antennal responses to EO compounds correlated with the outcomes of oviposition assays, except in the case of chamomile, where no antennal responses were detected. This may be due to the specificity of Q-fly antennae and palps towards certain chemicals; for instance, cuelure receptors are more abundant in Q-fly palps than in antennae (Park et al. 2018). Q-fly antennae may lack receptors for ­sesquiterpenes such as β-farnesene, α-bisabolol oxide A, or α-bisabolone oxide B, which are predominant compounds specifically found in chamomile. This highlights the critical importance of comprehending the chemical profiles of individual EOs and their interactions with insect sensory systems to optimize their use in pest management.

In conclusion, this study emphasizes the potential of EOs as sustainable tools for managing Q-fly populations. The variability in EO efficacy, driven by differences in chemical composition and application methods, highlights the necessity of optimizing formulations to enhance performance. The dose- and time-dependent nature of EO toxicities, along with sex-based differences in fumigation sensitivity, offers valuable insights for developing precise and effective pest control strategies. Furthermore, the importance of detailed chemical analyses is emphasized to ensure consistency and reliability in EO-based applications. This research provides a strong foundation for the development of targeted bioinsecticides, reducing dependence on synthetic chemicals and promoting environmentally sustainable agricultural practices.

## Supplementary Material

ieaf073_Supplementary_Data
