# Supplementary material for: Essential oils as potential insecticides and behavior-modifying agents against Bactrocera tryoni (Diptera: Tephritidae)
Source: J Insect Sci. 2025 Oct 6;25(5):ieaf073. doi: 10.1093/jisesa/ieaf073 (PMC12499782; doi:10.1093/jisesa/ieaf073)
Supplement: ieaf073_Supplementary_Data [file ieaf073_supplementary_data.docx]

Supplementary Material for the article

Essential oils as potential insecticides and behaviour-modifying agents against Queensland fruit fly *Bactrocera tryoni* (Diptera: Tephritidae)

Contents Page No.

Table S1. Contact toxicity assays. ED_50_ values and 95% confidence limits of EOs in male and female Q-fly. The values in mg represent the delivered amount of an EO when an aliquot of 1 μL of EO solution was applied on the dorsal thorax of adult Q-flies 2

Table S2. Fumigation toxicity assays. ED_50_ values and 95% confidence limits of EOs in male and female Q-fly. The values in μL/L air represent concentration of the delivered amount of an EO in a glass bottle... 3

Figure S1. Four-arm olfactometer bioassays. Average time per visit of Q-fly male in control and EO-treated zones. 4

Figure S2. Four-arm olfactometer bioassays. Average time per visit of Q-fly female in control and EO-treated zones. 4

Table S3. List of compounds in EOs analysed by GC-MS. The numbers in the column with EOs name in the table indicates the percentages of compounds (%) present in each EO. 5-9

References 10-12

**Table S1.** Contact toxicity assays. ED_50_ values and 95% confidence limits of EOs in male and female Q-fly after 6, 12 and 48 h of exposure. The values in mg represent the delivered amount of an EO when an aliquot of 1 μL of EO solution was applied on the dorsal thorax of adult Q-flies.

| Essential oil | Fly | 6 h exposure | | | | 12 h exposure | | | | 48 h exposure | | | |
| --- | --- | --- | --- | --- | --- | --- | --- | --- | --- | --- | --- | --- | --- |
|  |  | ED_50_  (mg/µL) | LCL | UCL | SE | ED_50_  (mg/µL) | LCL | UCL | SE | ED_50_  (mg/µL) | LCL | UCL | SE |
| Java citronella | Male | 0.098 | 0.080 | 0.119 | 0.080 | 0.073 | 0.057 | 0.089 | 0.078 | 0.051 | 0.039 | 0.064 | 0.077 |
|  | Female | 0.098 | 0.083 | 0.116 | 0.081 | 0.079 | 0.065 | 0.095 | 0.078 | 0.056 | 0.045 | 0.068 | 0.076 |
| Citronella grass | Male | 0.092 | 0.075 | 0.113 | 0.078 | 0.065 | 0.050 | 0.081 | 0.075 | 0.046 | 0.034 | 0.057 | 0.075 |
|  | Female | 0.108 | 0.091 | 0.127 | 0.084 | 0.084 | 0.069 | 0.100 | 0.08 | 0.060 | 0.049 | 0.072 | 0.077 |
| Lemon-scented tea tree | Male | 0.090 | 0.072 | 0.110 | 0.078 | 0.067 | 0.052 | 0.083 | 0.076 | 0.047 | 0.035 | 0.059 | 0.076 |
|  | Female | 0.104 | 0.088 | 0.122 | 0.082 | 0.084 | 0.069 | 0.100 | 0.079 | 0.058 | 0.047 | 0.070 | 0.077 |
| Aniseed | Male | 0.112 | 0.093 | 0.135 | 0.084 | 0.088 | 0.072 | 0.107 | 0.082 | 0.064 | 0.051 | 0.076 | 0.079 |
|  | Female | 0.114 | 0.097 | 0.134 | 0.086 | 0.096 | 0.08 | 0.113 | 0.083 | 0.071 | 0.059 | 0.083 | 0.080 |
| Yarrow | Male | 0.108 | 0.089 | 0.131 | 0.083 | 0.085 | 0.069 | 0.104 | 0.082 | 0.064 | 0.052 | 0.077 | 0.081 |
|  | Female | 0.115 | 0.097 | 0.135 | 0.087 | 0.100 | 0.084 | 0.118 | 0.085 | 0.077 | 0.065 | 0.090 | 0.082 |
| Pennyroyal | Male | 0.111 | 0.092 | 0.135 | 0.083 | 0.087 | 0.071 | 0.104 | 0.080 | 0.069 | 0.057 | 0.082 | 0.080 |
|  | Female | 0.107 | 0.091 | 0.126 | 0.083 | 0.089 | 0.074 | 0.106 | 0.080 | 0.069 | 0.057 | 0.082 | 0.079 |
| Thyme | Male | 0.113 | 0.094 | 0.137 | 0.085 | 0.088 | 0.072 | 0.107 | 0.083 | 0.065 | 0.053 | 0.078 | 0.081 |
|  | Female | 0.119 | 0.101 | 0.140 | 0.088 | 0.101 | 0.085 | 0.120 | 0.085 | 0.073 | 0.061 | 0.086 | 0.081 |
| Chamomile | Male | 0.086 | 0.069 | 0.106 | 0.077 | 0.066 | 0.051 | 0.082 | 0.076 | 0.048 | 0.036 | 0.06 | 0.076 |
|  | Female | 0.095 | 0.079 | 0.112 | 0.080 | 0.079 | 0.064 | 0.094 | 0.078 | 0.056 | 0.045 | 0.068 | 0.076 |
| LCL= Lower confidence level; UCL= Upper confidence level; SE= Standard error | | | | | | | | | | | | | |

**Table S2.** Fumigation toxicity assays. ED_50_ values and 95% confidence limits of EOs in male and female Q-fly after 6, 12 and 48 h of exposure. The values in μL/L air represent concentration of the delivered amount of an EO in a glass bottle.

| Essential oil | Fly | 6 h exposure | | | | 12 h exposure | | | | 48 h exposure | | | |
| --- | --- | --- | --- | --- | --- | --- | --- | --- | --- | --- | --- | --- | --- |
|  |  | ED_50_  (µL/L air) | LCL | UCL | SE | ED_50_  (µL/L air) | LCL | UCL | SE | ED_50_  (µL/L air) | LCL | UCL | SE |
| Aniseed | Male | 7.056 | 5.984 | 8.166 | 0.080 | 5.050 | 3.981 | 6.138 | 0.075 | 3.292 | 2.251 | 4.338 | 0.077 |
|  | Female | 7.807 | 6.773 | 8.874 | 0.084 | 4.915 | 3.948 | 5.897 | 0.077 | 3.655 | 2.68 | 4.640 | 0.078 |
| Basil | Male | 6.556 | 5.496 | 7.648 | 0.078 | 4.823 | 3.732 | 5.932 | 0.077 | 3.296 | 2.245 | 4.355 | 0.078 |
|  | Female | 7.278 | 6.270 | 8.317 | 0.083 | 5.626 | 4.64 | 6.632 | 0.079 | 4.217 | 3.217 | 5.232 | 0.081 |
| Citronella grass | Male | 10.509 | 9.156 | 11.928 | 0.102 | 8.603 | 7.354 | 9.904 | 0.091 | 6.058 | 4.956 | 7.185 | 0.087 |
|  | Female | 8.289 | 7.225 | 9.390 | 0.088 | 6.019 | 5.011 | 7.048 | 0.081 | 5.004 | 3.992 | 6.036 | 0.082 |
| Cumin | Male | 9.987 | 8.692 | 11.344 | 0.096 | 8.176 | 6.950 | 9.448 | 0.089 | 5.678 | 4.577 | 6.805 | 0.085 |
|  | Female | 8.267 | 7.202 | 9.370 | 0.087 | 5.713 | 4.720 | 6.726 | 0.084 | 4.210 | 3.224 | 5.211 | 0.081 |
| Dill | Male | 9.321 | 8.103 | 10.597 | 0.091 | 6.902 | 5.762 | 8.077 | 0.088 | 4.581 | 3.506 | 5.675 | 0.082 |
|  | Female | 7.776 | 6.750 | 8.836 | 0.085 | 5.617 | 4.627 | 6.626 | 0.080 | 3.669 | 2.679 | 4.67 | 0.08 |
| Garlic | Male | 5.774 | 4.758 | 6.815 | 0.077 | 3.830 | 2.774 | 4.892 | 0.075 | 2.739 | 1.701 | 3.776 | 0.077 |
|  | Female | 6.339 | 5.388 | 7.313 | 0.078 | 3.979 | 3.032 | 4.934 | 0.076 | 2.476 | 1.493 | 3.458 | 0.078 |
| Java citronella | Male | 10.741 | 9.384 | 12.169 | 0.099 | 8.136 | 6.931 | 9.389 | 0.088 | 6.284 | 5.19 | 7.405 | 0.085 |
|  | Female | 8.802 | 7.723 | 9.922 | 0.089 | 6.468 | 5.471 | 7.487 | 0.083 | 4.699 | 3.713 | 5.702 | 0.081 |
| Lemon-scented tea tree | Male | 9.691 | 8.442 | 11.000 | 0.092 | 8.009 | 6.82 | 9.244 | 0.085 | 5.432 | 4.375 | 6.509 | 0.081 |
|  | Female | 8.057 | 7.009 | 9.141 | 0.086 | 5.258 | 4.286 | 6.246 | 0.079 | 4.466 | 3.468 | 5.484 | 0.081 |
| Pennyroyal | Male | 8.264 | 7.107 | 9.468 | 0.086 | 5.394 | 4.284 | 6.526 | 0.079 | 4.377 | 3.305 | 5.466 | 0.081 |
|  | Female | 7.534 | 6.512 | 8.587 | 0.084 | 6.779 | 5.747 | 7.839 | 0.081 | 3.670 | 2.671 | 4.681 | 0.080 |
| Peppermint | Male | 7.819 | 6.705 | 8.976 | 0.083 | 5.970 | 4.858 | 7.109 | 0.080 | 3.932 | 2.869 | 5.007 | 0.080 |
|  | Female | 7.620 | 6.616 | 8.657 | 0.083 | 6.016 | 5.034 | 7.019 | 0.080 | 4.287 | 3.296 | 5.293 | 0.081 |
| River red gum | Male | 7.821 | 6.693 | 8.993 | 0.084 | 6.174 | 5.051 | 7.327 | 0.081 | 5.160 | 4.087 | 6.256 | 0.082 |
|  | Female | 8.953 | 7.836 | 10.113 | 0.092 | 6.892 | 5.825 | 7.988 | 0.086 | 5.682 | 4.635 | 6.756 | 0.086 |
| Tea tree | Male | 9.861 | 8.573 | 11.21 | 0.097 | 8.225 | 6.995 | 9.501 | 0.092 | 6.336 | 5.189 | 7.512 | 0.089 |
|  | Female | 9.999 | 8.817 | 11.231 | 0.097 | 8.014 | 6.935 | 9.125 | 0.088 | 5.900 | 4.874 | 6.947 | 0.086 |
| Thyme | Male | 8.017 | 6.864 | 9.216 | 0.086 | 6.610 | 5.448 | 7.808 | 0.082 | 4.843 | 3.742 | 5.965 | 0.082 |
|  | Female | 7.882 | 6.830 | 8.970 | 0.086 | 6.629 | 5.592 | 7.693 | 0.083 | 4.388 | 3.396 | 5.396 | 0.081 |
| Yarrow | Male | 9.766 | 8.494 | 11.099 | 0.096 | 7.825 | 6.618 | 9.075 | 0.089 | 6.115 | 5.000 | 7.259 | 0.085 |
|  | Female | 10.562 | 9.315 | 11.862 | 0.102 | 8.775 | 7.62 | 9.968 | 0.094 | 6.054 | 4.979 | 7.157 | 0.089 |
| LCL= Lower confidence level; UCL= Upper confidence level; SE= Standard error | | | | | | | | | | | | | |


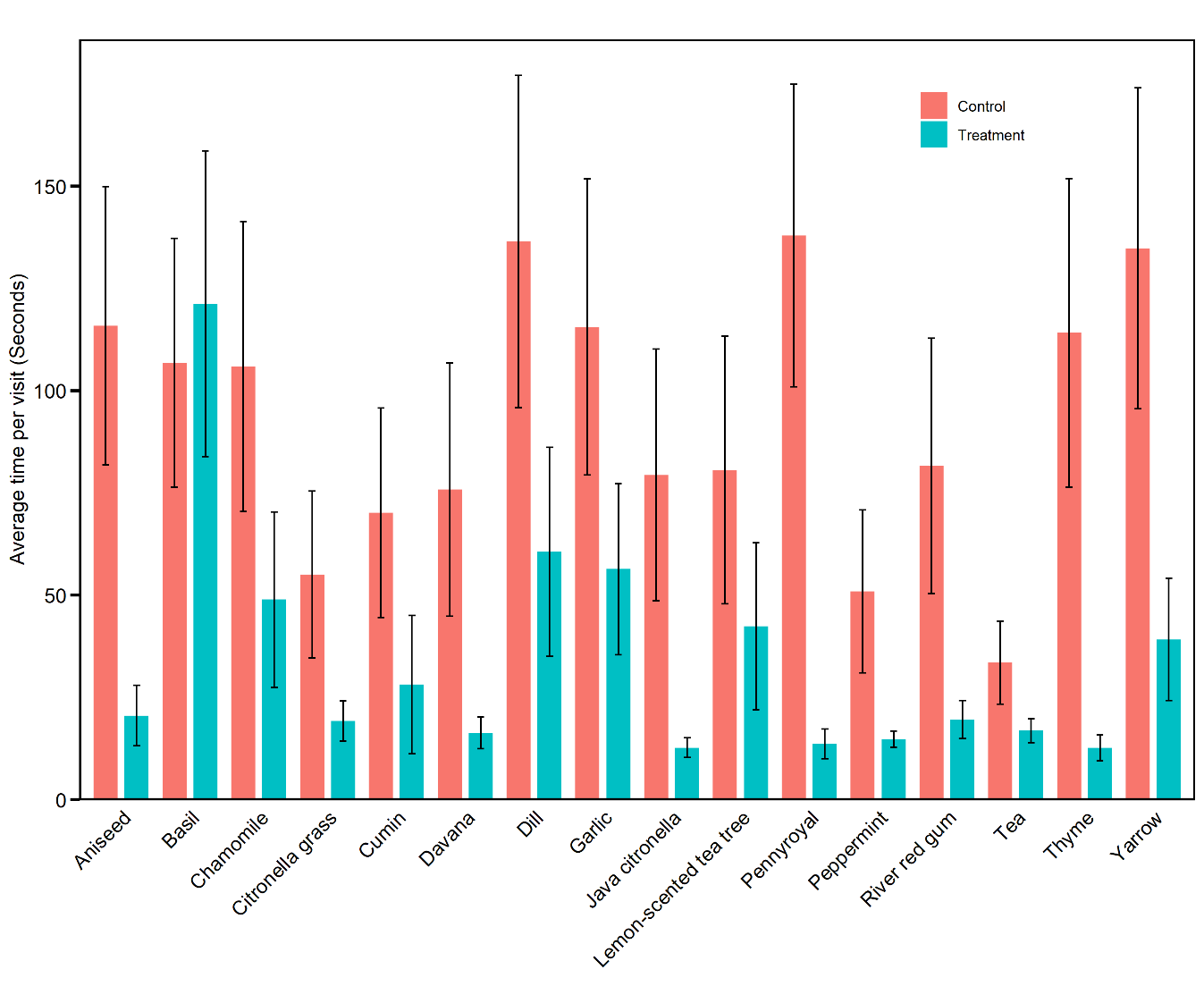


Figure S1. Four-arm olfactometer bioassays. Average time per visit of Q-fly male in control and EO-treated zones.


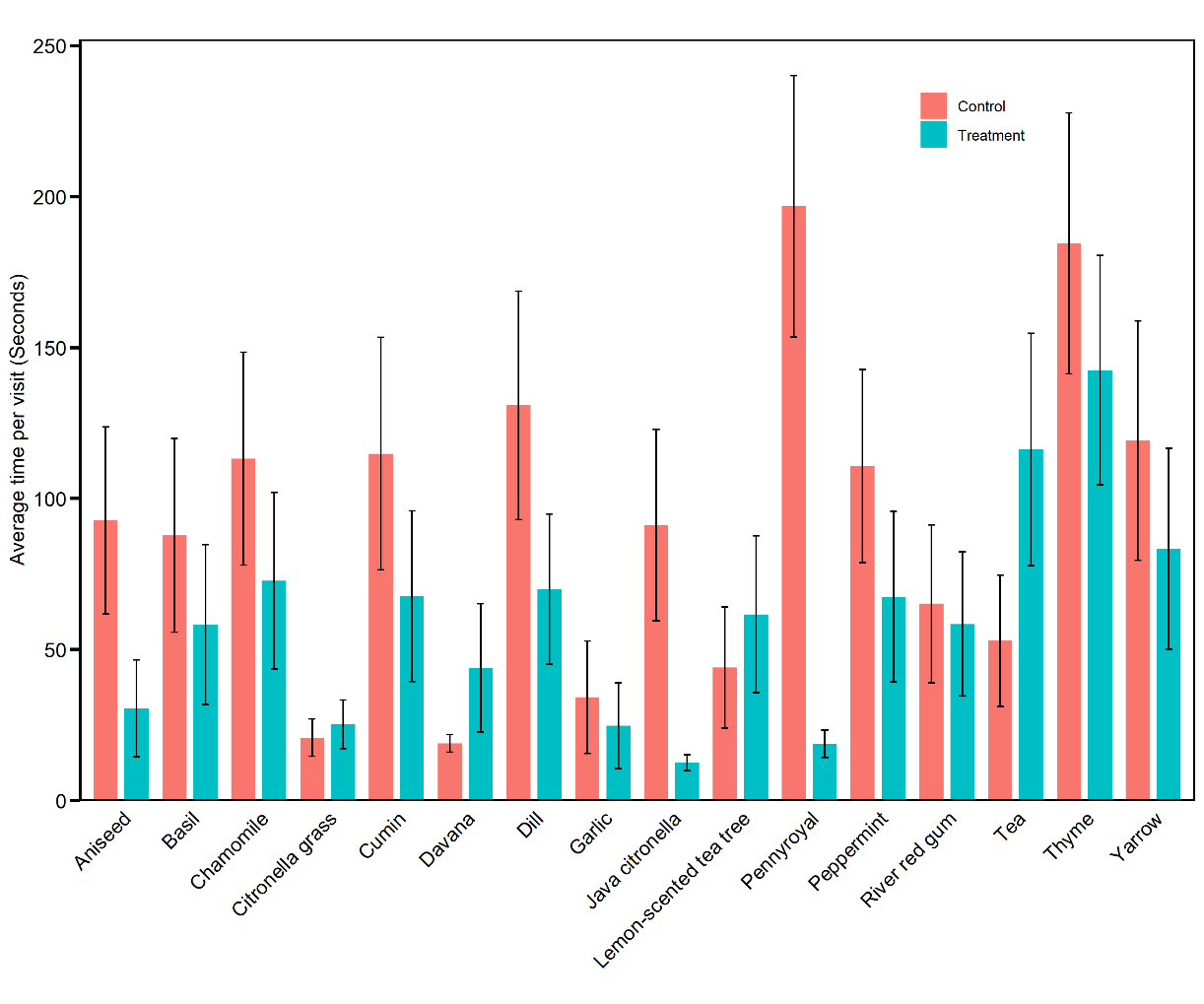


Figure S2. Four-arm olfactometer bioassays. Average time per visit of Q-fly female in control and EO-treated zones.

**Table S3:** List of compounds in EOs analysed by GC-MS. The numbers in the column with EOs name in the table indicate the percentages of compounds (%) present in each EO.

| Compound name | Retention Index (RI) | Dill | Java citronella | Peppermint | Cumin | Lemon-scented tea tree | Tea tree | Aniseed | Yarrow | Pennyroyal | Davana | Thyme | Citronella grass | Basil | Garlic | Chamomile | River red gum | Literature(s) identified the compound |
| --- | --- | --- | --- | --- | --- | --- | --- | --- | --- | --- | --- | --- | --- | --- | --- | --- | --- | --- |
| allyl methyl disulfide | 922 |  |  |  |  |  |  |  |  |  |  |  |  |  | 1.67 |  |  | Edris and Fadel 2002 |
| *α*-thujene | 931 |  |  |  |  |  | 0.74 |  |  |  |  |  |  |  |  |  |  | Brophy et al. 1989, Mondello et al. 1995, Shellie et al. 2003 |
| *α*-pinene | 941 |  |  | 1 |  | 0.86 | 2.02 | 2.99 | 12.14 | 0.71 |  | 0.95 |  |  |  |  | 1.99 | Shellie et al. 2003, Kocak et al. 2010,  Benayad et al. 2012, Yasa et al. 2012, Van Vuuren et al. 2014 |
| sabinene | 978 |  |  |  |  |  | 0.57 |  | 9.29 |  |  |  |  |  |  |  | 0.95 | Mondello et al. 1995, Shellie et al. 2003, Kocak et al. 2010, Ahmadi-Dastgerdi et al. 2017, Sahari et al. 2017 |
| 6-methyl-5-hepten-2-one | 984 |  |  |  |  |  |  |  |  |  |  |  |  |  |  |  |  |  |
| *β*-pinene | 985 |  |  | 1.41 | 5.31 | 0.82 | 0.72 |  | 10.04 | 0.53 |  | 0.69 |  |  |  |  | 0.77 | Tomescu et al. 2015, Buleandra et al. 2016, Moghadam 2016, Ahmadi-Dastgerdi et al. 2017 |
| *β*-myrcene | 990 | 0.59 |  |  |  | 0.65 | 0.86 | 1.44 | 0.82 |  |  | 0.39 |  |  |  |  | 0.85 | Debbab et al. 2007, Van Vuuren et al. 2014, Aćimović et al. 2015, Buleandra et al. 2016 |
| 2,3-dehydro-1,8-cineol | 995 |  |  |  |  | 0.32 |  |  |  |  |  |  |  |  |  |  |  |  |
| *α*-phellandrene | 1012 |  |  |  |  |  | 0.58 |  | 0.37 |  |  |  |  |  |  |  | 0.34 | Kocak et al. 2010, Mubarak et al. 2015, Sahari et al. 2017 |
| 1-methyl-4-(1-methylethyl)-1,3-cyclohexadiene | 1022 |  |  |  |  |  | 10.52 |  | 1.36 |  |  | 0.41 |  |  |  |  |  |  |
| *o-*cymene | 1025 |  |  | 0.54 |  |  |  |  |  |  |  |  |  |  |  |  |  |  |
| *p-*cymene | 1029 | 0.31 |  |  | 21.64 |  | 2.33 |  | 1.78 |  |  | 20.93 |  |  |  |  | 1.06 | Brophy et al. 1989, Mondello et al. 1995, Shellie et al. 2003, Ahmadi-Dastgerdi et al. 2017 |
| *D*-limonene | 1035 | 44.64 | 6.64 | 3.7 | 0.33 |  | 0.97 | 11.21 | 3.27 | 1.14 |  |  | 6.96 |  |  |  | 5.85 | Abena et al. 2007, Gende et al. 2009, Saleh-e-In et al. 2010, Ehsani and Mahmoudi 2012, Chen et al. 2014, Anastasopoulou et al. 2020, Hu et al. 2022 |
| eucalyptol | 1039 |  |  | 7.99 |  |  | 3.09 | 1.72 | 18.77 |  |  |  |  |  |  |  | 75.56 | Tomescu et al. 2015 |
| allyl 2-isopropyl disulfide | 1056 |  |  |  |  |  |  |  |  |  |  |  |  |  | 0.07 |  |  |  |
| artemisia ketone | 1059 |  |  |  |  |  |  |  |  |  |  |  |  |  |  | 1.1 |  |  |
| *γ*-terpinene | 1063 |  |  |  | 10.91 |  | 20.12 |  |  |  |  | 27.41 |  |  |  |  |  | Brophy et al. 1989, Mondello et al. 1995, Hudaib et al. 2002, Moghadam 2016 |
| *p*-mentha-3,8-diene | 1076 |  |  |  |  |  |  |  |  | 0.86 |  |  |  |  |  |  |  |  |
| diallyl disulfide | 1085 |  |  |  |  |  |  |  |  |  |  |  |  |  | 71.07 |  |  | Edris and Fadel 2002, Naher et al. 2014 |
| *α*-terpinolene | 1091 |  |  |  |  |  | 3.45 |  |  |  |  |  |  |  |  |  |  | Brophy et al. 1989, Mondello et al. 1995, Shellie et al. 2003 |
| (2-mehtyl-1-propenyl) bbenzene | 1095 |  |  |  |  |  |  |  |  |  |  |  |  |  |  |  |  |  |
| linalool | 1100 |  | 1.1 |  | 0.39 | 2.03 |  | 1.55 | 1.23 |  |  |  | 0.88 | 55.14 |  |  |  | Mahalwal and Ali 2003, Wesołowska et al. 2012, Chen et al. 2014, Avetisyan et al. 2017, Antić et al. 2019, Anastasopoulou et al. 2020, Hu et al. 2022, |
| allyl methyl trisulfide | 1148 |  |  |  |  |  |  |  |  |  |  |  |  |  | 0.59 |  |  | Edris and Fadel 2002 |
| citronellal | 1153 |  | 68.89 |  |  | 23.03 |  |  |  |  |  |  | 68.29 |  |  |  |  | Mahalwal and Ali 2003, Abena et al. 2007, Chen et al. 2014, Van Vuuren et al. 2014, Hu et al. 2022 |
| *p*-menth-3-en-8-ol | 1156 |  |  |  |  |  |  |  |  | 1.96 |  |  |  |  |  |  |  |  |
| (+)-2-bornanone | 1157 |  |  |  |  |  |  |  | 5.66 |  |  |  |  |  |  |  |  |  |
| trans-5-methyl-2-(1-methylethyl)-cyclohexanone (menthone) | 1163 |  |  | 32.84 |  |  |  |  |  | 0.46 |  |  |  |  |  |  |  |  |
| 4-methyl-1,2,3-trithiolane | 1171 |  |  |  |  |  |  |  |  |  |  |  |  |  | 1.21 |  |  |  |
| cis-5-methyl-2-(1-methylethyl)-cyclohexanone (isomenthone) | 1173 |  |  | 7.22 |  |  |  |  |  |  |  |  |  |  |  |  |  |  |
| isoborneol | 1174 |  |  |  |  |  |  |  | 2.45 |  |  |  |  |  |  |  |  | Ahmadi-Dastgerdi et al. 2017 |
| 3,7-dimethyl-3,6-octadienal | 1180 |  |  |  |  | 0.86 |  |  |  |  |  |  |  |  |  |  |  |  |
| neomenthol | 1182 |  |  | 1.21 |  |  |  |  |  | 1.87 |  |  |  |  |  |  |  |  |
| *l*-menthol | 1185 |  |  | 26.01 |  |  |  |  |  |  |  |  |  |  |  |  |  | Debbab et al. 2007, Tomescu et al. 2015, Buleandra et al. 2016 |
| 1-terpinen-4-ol | 1189 |  |  |  |  |  | 43.48 |  | 3.05 |  |  |  |  |  |  |  | 1.05 | Brophy et al. 1989, Shellie et al. 2003, Mubarak et al. 2015 |
| *α*-terpineol | 1202 |  |  |  |  |  | 2.12 |  | 3.12 |  |  |  |  |  |  |  | 3.65 | Brophy et al. 1989, Mondello et al. 1995, Shellie et al. 2003, Kocak et al. 2010, Mubarak et al. 2015 |
| estragole (methyl chavicol) | 1203 |  |  |  |  |  |  |  |  |  |  |  |  | 44.86 |  |  |  | Wesołowska et al. 2012, Joshi 2014,  Avetisyan et al. 2017, Antić et al. 2019 |
| dihydrocarvone diastereomer 1 | 1204 | 2.72 |  |  |  |  |  |  |  |  |  |  |  |  |  |  |  | Saleh-E-In and Choi 2021 |
| dihydrocarvone diastereomer 2 | 1212 | 1.97 |  |  |  |  |  |  |  |  |  |  |  |  |  |  |  | Saleh-E-In and Choi 2021 |
| citronellole | 1241 |  | 0.66 |  |  |  |  |  |  |  |  |  |  |  |  |  |  |  |
| neral | 1242 |  | 0.66 |  |  | 29.75 |  |  |  |  |  |  | 0.85 |  |  |  |  | Abena et al. 2007, Van Vuuren et al. 2014 |
| pulegone | 1247 |  |  | 0.59 |  |  |  |  |  | 92.77 |  |  |  |  |  |  |  | Debbab et al. 2007, Mahboubi and Haghi 2008, Benayad et al. 2012, Yasa et al. 2012, Tomescu et al. 2015, Buleandra et al. 2016 |
| linalyl acetate | 1250 |  |  |  |  |  |  |  | 1.7 |  |  |  |  |  |  |  |  |  |
| *d*-carvone | 1251 | 40.48 |  |  |  |  |  |  |  |  |  |  |  |  |  |  |  |  |
| *p-*isopropylbenzaldehyde (cuminaldehyde) | 1251 |  |  |  | 54.1 |  |  |  |  |  |  |  |  |  |  |  |  |  |
| *p*-methoxybenzaldehyde | 1263 |  |  |  |  |  |  | 0.76 |  |  |  |  |  |  |  |  |  |  |
| geranial | 1270 |  | 1.14 |  |  | 42.17 |  |  |  |  |  |  | 2.59 |  |  |  |  | Mahalwal and Ali 2003, Abena et al. 2007, Van Vuuren et al. 2014 |
| thymol | 1291 |  |  |  |  |  |  |  |  |  |  | 28.39 |  |  |  |  |  | Hudaib et al. 2002 |
| (*E*)- anethole | 1293 |  |  |  |  |  |  | 80.33 |  |  |  |  |  |  |  |  |  | Kurkcuoglu et al. 2003, Tabanca et al. 2006, Gende et al. 2009, Aćimović et al. 2015, Anastasopoulou et al. 2020, |
| menthyl acetate | 1294 |  |  | 7.99 |  |  |  |  |  |  |  |  |  |  |  |  |  | Debbab et al. 2007, Buleandra et al. 2016 |
| isobornyl acetate | 1295 |  |  |  |  |  |  |  | 5.58 |  |  |  |  |  |  |  |  | Sahari et al. 2017 |
| eucarvone | 1296 |  |  |  | 6.17 |  |  |  |  |  |  |  |  |  |  |  |  |  |
| di-2-propenyl trisulfide | 1313 |  |  |  |  |  |  |  |  |  |  |  |  |  | 20.36 |  |  | Naher et al. 2014 |
| citronellyl acetate | 1348 |  | 3.62 |  |  |  |  |  |  |  |  |  | 3.34 |  |  |  |  | Abena et al. 2007 |
| *α*-terpinyl acetate | 1353 |  |  |  |  |  |  |  |  |  |  |  |  |  |  |  | 0.44 |  |
| nerol acetate | 1358 |  |  |  |  |  |  |  | 4.01 |  |  |  |  |  |  |  |  |  |
| geranyl acetate | 1376 |  | 5.26 |  |  |  |  |  |  |  |  |  | 8.45 |  |  |  |  | Abena et al. 2007, Chen et al. 2014, Hu et al. 2022 |
| elemene | 1400 |  | 3.21 |  |  |  |  |  |  |  |  |  | 1.63 |  |  |  |  | Mahalwal and Ali 2003, Abena et al. 2007, Chen et al. 2014, Hu et al. 2022, |
| 1a,2,3,3a,4,5,6,7b-octahydro-1,1,3a,7-tetramethyl-1H-Cyclopropa[a]naphthalene | 1424 |  |  |  |  |  | 0.24 |  |  |  |  |  |  |  |  |  |  |  |
| *β* -caryophylene | 1438 |  | 0.8 | 2.63 |  |  | 0.29 |  | 6.52 |  |  |  | 1.17 |  |  |  |  | Brophy et al. 1989, Sahari et al. 2017 |
| *β*-farnesene | 1455 |  |  |  |  |  |  |  |  |  |  |  |  |  |  | 53.09 |  | Ayoughi et al. 2011 |
| aromandendrene | 1457 |  |  |  |  |  | 0.87 |  |  |  | 1.11 |  |  |  |  |  |  | Brophy et al. 1989, Shellie et al. 2003, Bail et al. 2008 |
| ethyl cinnamate | 1474 |  |  |  |  |  |  |  |  |  | 6.27 |  |  |  |  |  |  | Bail et al. 2008 |
| alloaromadendrene | 1479 |  |  |  |  |  | 0.5 |  |  |  |  |  |  |  |  |  |  |  |
| davana ether | 1486 |  |  |  |  |  |  |  |  |  | 2.24 |  |  |  |  |  |  | Bail et al. 2008 |
| (1R,4R,5S)-1,8-Dimethyl-4-(prop-1-en-2-yl)spiro[4.5]dec-7-ene | 1492 |  |  |  | 1.88 |  |  |  |  |  |  |  |  |  |  |  |  |  |
| germacreneD | 1498 |  | 3.49 |  |  |  |  |  |  |  |  |  | 0.83 |  |  | 2.56 |  | Abena et al. 2007,  Chen et al. 2014, Stanojevic et al. 2016 |
| davana ether isomer | 1506 |  |  |  |  |  |  |  |  |  | 5.47 |  |  |  |  |  |  | Bail et al. 2008 |
| *α*-farnesene | 1507 |  |  |  |  |  |  |  |  |  |  |  |  |  |  | 2.39 |  | Ayoughi et al. 2011 |
| 1,2,3,5,6,7,8,8a-octahydro-1,4-dimethyl-7-(1-methylethenyl)-azulene | 1508 |  |  |  |  |  | 1.22 |  |  |  |  |  |  |  |  |  |  |  |
| bicyclogermacrene | 1513 |  |  |  |  |  | 0.97 |  |  |  | 12.08 |  |  |  |  | 1.61 |  | Brophy et al. 1989, Shellie et al. 2003, Bail et al. 2008, Stanojevic et al. 2016 |
| *δ*-cadinene | 1531 |  | 4.73 |  |  |  | 0.92 |  |  |  |  |  | 3.05 |  |  |  |  | Brophy et al. 1989, Shellie et al. 2003, Abena et al. 2007, Chen et al. 2014, Tomescu et al. 2015 |
| zonarene | 1538 |  |  |  |  |  | 0.3 |  |  |  |  |  |  |  |  |  |  |  |
| germacreneB | 1548 |  |  |  |  |  | 0.25 |  |  |  |  |  |  | 0.77 |  |  |  |  |
| di-2-propenyl tetrasulfide | 1560 |  |  |  |  |  |  |  |  |  |  |  |  |  | 4.56 |  |  |  |
| elemol | 1562 |  | 0.92 |  |  |  |  |  |  |  |  |  | 1.96 |  |  |  |  | Abena et al. 2007, Chen et al. 2014, Hu et al. 2022 |
| davanone | 1585 |  |  |  |  |  |  |  |  |  | 87.93 |  |  |  |  |  |  | Bail et al. 2008 |
| diethyl phthalate | 1591 |  |  |  |  |  |  |  |  |  |  |  |  |  |  |  | 0.97 |  |
| apiole | 1627 | 9.6 |  |  |  |  |  |  |  |  |  |  |  |  |  |  |  | Saleh-e-In et al. 2010, 2017, Saleh-E-In and Choi 2021 |
| methyl (3-oxo-2-pentylcyclopentyl) acetate | 1654 |  |  |  |  |  |  |  | 3.94 |  |  |  |  |  |  |  |  |  |
| *τ*-cadinol | 1659 |  |  |  |  |  |  |  |  |  |  |  |  |  |  | 0.98 |  |  |
| *α*-bisabolol oxide B | 1671 |  |  |  |  |  |  |  |  |  |  |  |  |  |  | 10.78 |  | Stanojevic et al. 2016 |
| (+)-methyldihydroepiasmonate | 1683 |  |  |  |  |  |  |  | 0.48 |  |  |  |  |  |  |  |  |  |
| α-bisabolone oxide A | 1699 |  |  |  |  |  |  |  |  |  |  |  |  |  |  | 18.98 |  |  |
| 1,2,3,4-teteamethyl naphthalene | 1754 |  |  |  |  |  |  |  |  |  |  |  |  |  |  | 5.29 |  |  |
| isopropyl myristate | 1822 |  |  |  |  |  |  |  |  |  |  |  |  |  |  |  | 6.52 |  |
| 1-allyl-3-(2-(allylthio) propyl) trisulfane | 1838 |  |  |  |  |  |  |  |  |  |  |  |  |  | 4.01 |  |  |  |
| 2-isopropyl-3-methylnaphthalen-1(2*H*)-one | 1895 |  |  |  |  |  |  |  |  |  |  |  |  |  |  | 5.4 |  |  |

**References**

Abena AA, Gbenou JD, Yayi E, et al. 2007. Comparative chemical and analgesic properties of essential oils of *Cymbopogon nardus* (L) Rendle of Benin and Congo. Afr. J. Tradit. Compl. Altern. Med. 4(3):267–272. <https://doi.org/10.4314/ajtcam.v4i3.31218>

Aćimović MG, Tešević V, Todosijević M, et al. 2015. Compositional characteristics of the essential oil of *Pimpinella anisum* and *Foeniculum vulgare* grown in Serbia. Bot. Serbica. 39(1):9–14. <https://hdl.handle.net/21.15107/rcub_cherry_286>

Ahmadi-Dastgerdi A, Ezzatpanah H, Asgary S, et al. 2017. Phytochemical, Antioxidant and Antimicrobial Activity of the Essential Oil from Flowers and Leaves of *Achillea millefolium* subsp. *millefolium*. J. Essent. Oil-Bear. Plants. 20(2):395–409. <https://doi.org/10.1080/0972060X.2017.1280419>

Anastasopoulou E, Graikou K, Ganos C, et al. 2020. *Pimpinella anisum* seeds essential oil from Lesvos island: Effect of hydrodistillation time, comparison of its aromatic profile with other samples of the Greek market. Safe use. Food Chem. Toxicol. 135:110875. <https://doi.org/10.1016/j.fct.2019.110875>

Antić MP, Jelačić SC, Knudsen TM. 2019. Chemical composition of the essential oils of three *Ocimum basilicum* L. cultivars from Serbia. Not. Bot. Horti. Agrobot. 47(2):347–351. <https://doi.org/10.15835/nbha47111250>

Avetisyan A, Markosian A, Petrosyan M, et al. 2017. Chemical composition and some biological activities of the essential oils from *Basil ocimum* different cultivars. BMC Complement Altern. Med. 17(1):1–8. <https://doi.org/10.1186/s12906-017-1587-5>

Ayoughi F, Marzegar M, Sahari MA, et al. 2011. Chemical compositions of essential oils of *Artemisia dracunculus* L. and endemic *Matricaria chamomilla* L. and an evaluation of their antioxidative effects. J. Agric. Sci. Technol. 13(1):79–88. <http://dorl.net/dor/20.1001.1.16807073.2011.13.1.13.6>

Bail S, Buchbauer G, Schmidt E, et al. 2008. GC-MS-analysis, antimicrobial activities and olfactory evaluation of essential davana (*Artemisia pallens* Wall. ex DC) oil from India. Nat. Prod. Commun. 3(7):1934578X0800300705. <https://doi.org/10.1177/1934578X0800300705>

Benayad N, Ebrahim W, Hakiki A, et al. 2012. Chemical characterization and insecticidal evaluation of the essential oil of *Mentha suaveolens* L. and *Mentha pulegium* L. growing in Morocco. Sci. Stud. Res., Chem. Chem. Eng. Biotechnol. Food Ind 13(1):27.

Brophy JJ, Davies NW, Southwell IA, et al. 1989. Gas chromatographic quality control for oil of *Melaleuca* terpinen-4-ol type (Australian tea tree). J. Agric. Food Chem. 37(5):1330–1335. <https://doi.org/10.1021/jf00089a027>

Buleandra M, Oprea E, Popa DE, et al. 2016. Comparative chemical analysis of *Mentha piperita* and M*. spicata* and a fast assessment of commercial peppermint teas. Nat. Prod. Commun. 11(4):1934578X1601100433. <https://doi.org/10.1177/1934578X1601100433>

Chen Q, Xu S, Wu T, et al. 2014. Effect of citronella essential oil on the inhibition of postharvest *Alternaria alternata* in cherry tomato. J. Sci. Food Agric. 94(12):2441–2447. <https://doi.org/10.1002/jsfa.6576>

Debbab A, Mosaddak B, Aly AH, et al. 2007. Chemical characterization and toxicological evaluation of the essential oil of *Mentha piperita* L. growing in Morocco. Sci. Stu. Resource. 8(3):281–288.

Edris AE, Fadel HM. 2002. Investigation of the volatile aroma components of garlic leaves essential oil. Possibility of utilization to enrich garlic bulb oil. Eur. Food Res. Technol. 214(2):105–107. <https://doi.org/10.1007/s00217-001-0429-2>

Ehsani A, Mahmoudi R. 2012. Phytochemical properties and hygienic effects of *Allium ascalonicum* and *Pimpinella anisum* essential oils in Iranian white brined cheese. J. Essent. Oil-Bear. Plants. 15(6):1013–1020. <https://doi.org/10.1080/0972060X.2012.10662606>

Gende LB, Maggi MD, Fritz R, et al. 2009. Antimicrobial activity of *Pimpinella anisum* and *Foeniculum vu*lgare essential oils against *Paenibacillus* larvae. J. Essent. Oil Res. 21(1):91–93. <https://doi.org/10.1080/10412905.2009.9700120>

Hu HL, Zhou D, Wang JW, et al. 2022. Chemical composition of citronella (*Cymbopogon winterianus*) leaves essential oil and gastric toxicity of its major components to *Drosophila melanogaster* larvae. J. Essent. Oil-Bear. Plants. 25:1–1. <https://doi.org/10.1080/0972060X.2022.2077142>

Hudaib M, Speroni E, Di Pietra AM, et al. 2002. GC/MS evaluation of thyme (*Thymus vulgaris* L.) oil composition and variations during the vegetative cycle. J. Pharm. Biomed. Anal. 29(4):691–700. <https://doi.org/10.1016/S0731-7085(02)00119-X>

Joshi RK. 2014. Chemical composition and antimicrobial activity of the essential oil of *Ocimum basilicum* L. (sweet basil) from Western Ghats of North West Karnataka, India. Anc. Sci. Life. 33(3):151–156. <https://doi.org/10.4103/0257-7941.144618>

Kocak A, Bagci E, Bakoglu A. 2010. Chemical composition of essential oils of *Achillea teretifoli*a Willd. and *A. millefolium* L. subsp. *millefolium* growing in Turkey. Asian J. Chem. 22(5):3653–3658.

Kurkcuoglu M, Sargin N, Baser KH. 2003. Composition of volatiles obtained from spices by microdistillation. Chem. Nat. Compd. 39(4):355–357. <https://doi.org/10.1023/B:CONC.0000003415.49701.8b>

Mahalwal VS, Ali M. 2003. Volatile constituents of *Cymbopogon nardus* (Linn.) Rendle. Flav. Fragr. J. 18:73–76. <https://doi.org/10.1002/ffj.1144>

Mahboubi M, Haghi G. 2008. Antimicrobial activity and chemical composition of *Mentha pulegium* L. essential oil. J. Ethnopharmacol. 119(2):325–327. <https://doi.org/10.1016/j.jep.2008.07.023>

Moghadam AL. 2016. Efficacy of chemically characterized *Cuminum cyminum* essential oil as an antioxidant and lipid peroxidation inhibitor. J. Essent. Oil-Bear. Plants. 19(1):134–139. <https://doi.org/10.1080/0972060X.2015.1086284>

Mondello L, Dugo P, Basile A, et al. 1995. Interactive use of linear retention indices, on polar and apolar columns, with a MS‐library for reliable identification of complex mixtures. J. Microcolumn. Sep. 7(6):581–591. <https://doi.org/10.1002/mcs.1220070605>

Mubarak EE, Landa ZA, Ahmed IF, et al. 2015. Essential oil compositions and cytotoxicity from various organs of *Eucalyptus camaldulensis*. Int. J. Agric. Biol. Apr. 17(2).

Naher S, Ahmad MM, Aziz S, et al. 2014. Comparative studies on physicochemical properties and GC-MS analysis of essential oil of two varieties of *Allium sativum* Linn (garlic). Int. J. Pharm. Phytopharmacological Res. 4(3):173–175.

Sahari MA, Mehrafarin A, Naghdi BH. 2017. Chemical composition and antioxidant activity *Achillea millefolium* L. essential oils. J. Essent. Oil-Bear. Plants. 20(1):293–297. <https://doi.org/10.1080/0972060X.2016.1238323>

Saleh-E-In MM, Choi YE. 2021. *Anethum sowa* Roxb. ex fleming: A review on traditional uses, phytochemistry, pharmacological and toxicological activities. J. Ethnopharm. 280:113967. <https://doi.org/10.1016/j.jep.2021.113967>

Saleh-e-In MM, Sultana A, Husain M, et al. 2010. Chemical constituents of essential oil from *Anethum sowa* L. herb (leaf and stem) growing in Bangladesh. Bangladesh J. Sci. Ind. Res. 45(2):173-176. <http://dx.doi.org/10.3;329/bjsir.v45i2.5721>

Saleh-e-In MM, Sultana N, Rahim MM, et al. 2017. Chemical composition and pharmacological significance of *Anethum Sowa* L. Root. BMC Complement Altern. Med. 17(1):1–7.

Shellie R, Marriott P, Zappia G, et al. 2003. Interactive use of linear retention indices on polar and apolar columns with an MS-library for reliable characterization of Australian tea tree and other *Melaleuca* sp. oils. J. Essent. Oil Res. 15(5):305–312. <https://doi.org/10.1080/10412905.2003.9698597>

Stanojevic LP, Marjanovic-Balaban ZR, Kalaba VD, et al. 2016. Chemical composition, antioxidant and antimicrobial activity of chamomile flowers essential oil (*Matricaria chamomilla* L.). J. Essent. Oil-Bear. Plants. 19(8):2017–2028. <https://doi.org/10.1080/0972060X.2016.1224689>

Tabanca N, Demirci B, Ozek T, et al. 2006. Gas chromatographic–mass spectrometric analysis of essential oils from *Pimpinella* species gathered from Central and Northern Turkey. J. Chromatogr. A. 1117(2):194–205. <https://doi.org/10.1016/j.chroma.2006.03.075>

Tomescu A, Sumalan RM, Pop G, et al. 2015. Chemical composition and protective antifugal activity of *Mentha piperita* L. and *Salvia officinalis* L. essential oils against *Fusarium graminearum* spp. Rev. Chim. 66(7):1027–1030.

Van Vuuren SF, Docrat Y, Kamatou GP, et al. 2014. Essential oil composition and antimicrobial interactions of understudied tea tree species. South Afr. J. Bot. 92:7–14. <https://doi.org/10.1016/j.sajb.2014.01.005>

Wesołowska A, Kosecka D, Jadczak D. 2012. Essential oil composition of three sweet basil (*Ocimum basilicum*) cultivars. Herba. Polonica. 58(2):516.

Yasa H, Onar HÇ, Yusufoglu AS. 2012. Chemical composition of the essential oil of *Mentha pulegium* L. from Bodrum, Turkey. J. Essent. Oil-Bear Plants. 15(6):1040–1043. <https://doi.org/10.1080/0972060X.2012.10662609>
